# Structural and Biochemical Characterization of AaL, a Quorum Quenching Lactonase with Unusual Kinetic Properties

**DOI:** 10.1038/s41598-018-28988-5

**Published:** 2018-07-26

**Authors:** Celine Bergonzi, Michael Schwab, Tanushree Naik, David Daudé, Eric Chabrière, Mikael Elias

**Affiliations:** 1 Biochemistry, Molecular Biology & Biophysics Dpt and BioTechnology Institute, University of Minnesota, Saint Paul, Minnesota 55108 USA; 2 Gene&GreenTK, 19–21 Boulevard Jean Moulin, 13005 Marseille, France; 3 Aix Marseille Univ, IRD, APHM, MEPHI, IHU-Méditerranée Infection, Marseille, France

## Abstract

Quorum quenching lactonases are enzymes that are capable of disrupting bacterial signaling based on acyl homoserine lactones (AHL) *via* their enzymatic degradation. In particular, lactonases have therefore been demonstrated to inhibit bacterial behaviors that depend on these chemicals, such as the formation of biofilms or the expression of virulence factors. Here we characterized biochemically and structurally a novel representative from the metallo-β-lactamase superfamily, named AaL that was isolated from the thermoacidophilic bacterium *Alicyclobacillus acidoterrestris*. AaL is a potent quorum quenching enzyme as demonstrated by its ability to inhibit the biofilm formation of *Acinetobacter baumannii*. Kinetic studies demonstrate that AaL is both a proficient and a broad spectrum enzyme, being capable of hydrolyzing a wide range of lactones with high rates (k_cat_/K_M_ > 10^5^ M^−1^.s^−1^). Additionally, AaL exhibits unusually low K_M_ values, ranging from 10 to 80 µM. Analysis of AaL structures bound to phosphate, glycerol, and C6-AHL reveals a unique hydrophobic patch (W26, F87 and I237), involved in substrate binding, possibly accounting for the enzyme’s high specificity. Identifying the specificity determinants will aid the development of highly specific quorum quenching enzymes as potential therapeutics.

## Introduction

Bacterial quorum sensing (QS) is one of the most prominent communication system displayed by bacteria. This system depends on the production and detection of signaling molecules referred to as “autoinducers” to coordinate gene expression in response to cell density. A common class of autoinducers are *N*-acyl-L-homoserine lactones (AHLs)^1^. AHL-based bacterial quorum sensing was shown to regulate various behaviors in numerous microbes, mainly gram-negatives, including virulence factors production and biofilm formation^2^. Some enzymes, named Quorum Quenching (QQ) enzymes, are naturally capable of interfering with AHL-based QS, via the enzymatic modification or degradation of the signaling molecules^3^. Consequently, these enzymes were previously reported as biofilm and virulence inhibitors^4^ during *in vitro* and *in vivo* experiments^5–8^. They comprise promising candidates as potential protein therapeutics^9^, to prevent infection in livestocks^10^ and to prevent biofouling^11^.

AHL-degrading enzymes can be found in organisms beyond the bacterial world, e.g. archae, plants, fungi and mammals^12,13^. Some of the most widely used and studied QQ enzymes are lactonases. Lactonases were identified and characterized from three main protein superfamilies^14^. The paraoxonases (PONs) are lactonases that were primarily identified in mammals^13,15,16^. PONs adopt a six-bladed β-propeller fold and a central tunnel with two calcium cations^17,18^, one being structural, the second being involved in catalysis^19^. PONs were shown to hydrolyze δ-lactones, γ-lactones and AHLs^20^. Another class of lactonases is the Phosphotriesterases-like lactonase (PLL). Several PLLs were identified in several extremophiles^21–23^ and accordingly exhibit remarkable thermal stability (up to 128 °C for VmoLac^22^) and high rates for lactone hydrolysis^22^. PLLs were divided into two subclasses, A and B, where PLL-As can hydrolyze δ-lactones, γ-lactones and AHLs and PLL-Bs prefer δ-lactones and γ-lactones^22^. Notably, certain γ-lactones can be used as QS molecules in *Streptomyces* and *Rhodococcus*^24,25^.

The metallo-β-lactamase like (MLLs) or AiiA-like represents another class of lactonases. The most characterized enzyme from this family is the autoinducer inactivator A (AiiA) from *Bacillus thuringiensis*^26^, but other representatives have been studied, such as AiiB^27^, AidC^28^, MomL^29^ or GcL^30^. The MLLs enzymes possess the conserved motif “HXHXDH” that allows for the formation of the bi-metallic active site. Its crystal structure has been solved^31^ and its catalytic mechanism has been investigated^26,32^. Numerous experiments *in vivo* and *in vitro* showed the efficiency of these enzymes to degrade the quorum sensing signals, through the inhibitions of virulence factor production or biofilm synthesis for examples^3,33^. MLLs, including AiiA, AidC, MomL, were reported to exhibit broad substrate range with respect to the acyl chain length of AHLs^4^, but their potential activity on δ-lactones or γ-lactones is unknown.

**Figure 1:**
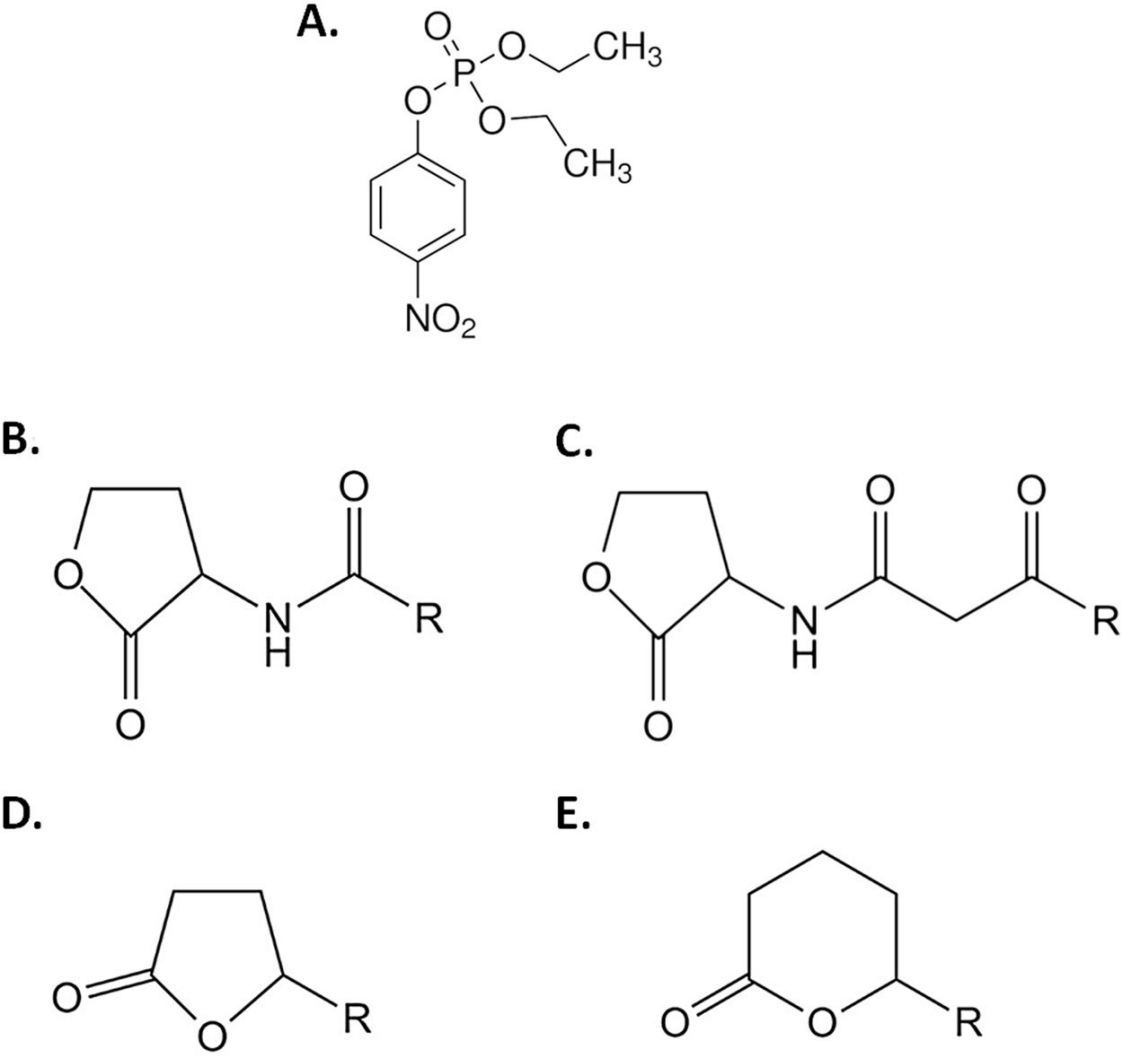
Structures of the tested chemicals. (**A**) Paraoxon-ethyl (**B**) Acyl-Homoserine Lactones, (**C**) 3-oxo-Acyl-Homoserine Lactones, (**D**) γ-lactones and (**E**) δ-lactones

AaL (WP_021296945.1) is a lactonase isolated from the acidophilic, moderately thermostable bacterium *Alicyclobacillus acidoterrestris*. Consequently, AaL is moderately thermostable, with a T_m_ of 58.2 °C^34^. It shares 27% sequence identity with AiiA and 43% sequence identity with AiiB. Previous kinetic studies on AaL revealed that it is a very proficient lactonase, with broad specificity spectrum, and also that it exhibits unusually low K_M_ values as compared to other MLLs lactonases^34^. Indeed, K_M_ values for AaL were found to range between 10 to 83 µM^34^ with AHLs as substrates, while several other MLLs were reported to exhibit much higher K_M_ values (~1 mM for AiiA^26,32^, 440 µM for MomL^29^) with the exception of AidC (46–72 μM^35^). In AidC, its high specificity was attributed to atypical structural features in the vicinity of the active site^28^. Other classes of lactonases (e.g. PONs and PLLs) were reported to possess relatively low K_M_ values (approximately 50 to 500 μM^20,22,36,37^) (Table 1). Low K_M_ values enzymes are of particular interest because their study may allow for a better understanding on the structural determinants for lactones binding, and may represent promising tools and targets for their optimization as therapeutic, anti-virulence and anti-biofilm enzymes. Therefore, we undertook the structural characterization of the purified AaL. We show that AaL is capable of degrading δ-lactones and γ-lactones with high catalytic proficiency (>10^4^ M^−1^ s^−1^) and shows some weak, promiscuous phosphotriesterase activity on the insecticide-derivated paraoxon (Fig. 1). The crystal structure of AaL reveals a unique hydrophobic patch which could relate to its high specificity, and highlights a flexible active site loop which may be involved in substrate binding.

**Table 1:** Kinetics parameters for several quorum quenching enzyme representatives.

Enzyme		Organism	Substrate	K_cat_ (s^−1^)	K_M_ (µM)	K_cat_/K_M_ (M^−1^ s^−1^)	Reference
MBLs	AaL	*Alicyclobacillus acidoterrestris*	C4-AHL	12.6	9	1.3 × 10^6^	This work*
	AiiA	*Bacillus thuringiensis*	C6-AHL	91	5600	1.6 × 10^4^	^26^
	AiiB	*Agrobacterium tumefaciens*	C6-AHL	24.8	1600	1.6 × 10^4^	^27^
	AidC	*Chryseobacterium* sp. strain StRB126	C7-AHL	80	47	1.7 × 10^6^	^28^
	MomL	*Muricauda olearia*	3-oxo-C10-AHL	224	440	5.1 × 10^5^	^29^
PONs	PON2	Mammals	3-oxo-C12-AHL	13	50	2.7 × 10^5^	^20^
	PON-OCCAL	Mammals	3-oxo-C12-AHL	44.5	180	2.4 × 10^5^	^20^
PLLs	SsoPox	*Sulfolobus solfataricus*	3-oxo-C10-AHL	4.5	143	3.6 × 10^4^	^36^
	VmoLac	*Vulcanisaeta moutnovskia*	3-oxo-C10-AHL	0.5	231	2.1 × 10^3^	^22^
	SacPox	*Sulfolobus acidocaldarius*	C8-AHL	0.9	178	5.3 × 10^3^	^77^
	SisLac	*Sulfolobus islandicus*	3-oxo-C8-AHL	4.1	42	9.7 × 10^4^	^23^
Acylase	PvdQ	*Pseudomonas aeruginosa*	C12-AHL	2.5	11	2.2 × 10^5^	^78^

*The kinetics were performed in *activity buffer* (2.5 mM Bicine pH 8.3, 150 mM NaCl, 0.2 mM cresol purple, 0.5**%** DMSO and with 0.2 mM CoCl_2_).

## Results and Discussion

### Sequence analysis

**Figure 2:**
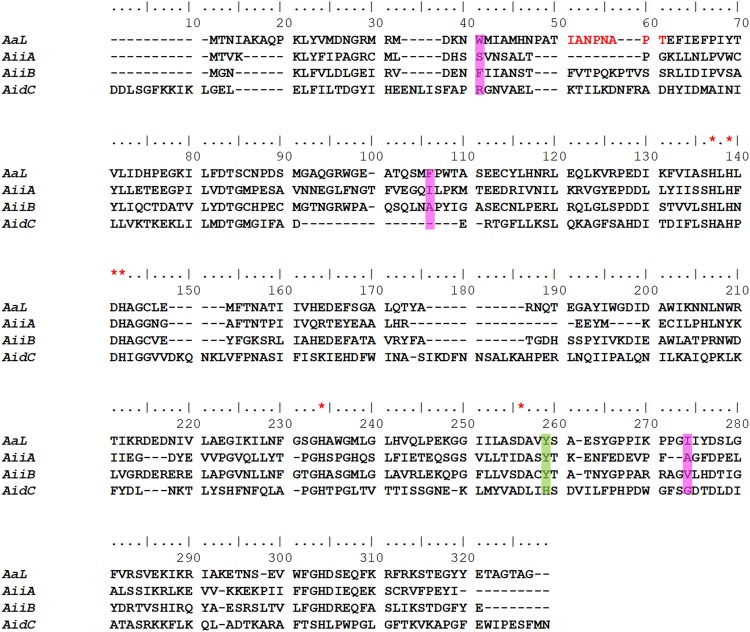
Sequence alignment of the MLLs representatives. Sequence alignment of AaL from Alicyclobacillus acidoterrestris (WP_021296945.1), AiiA from Bacillus thuringiensis (PMC1187999), AiiB from Agrobacterium tumefaciens (WP_010974862.1) and AidC from Chryseobacterium sp. StRB126 (PMC4681436). The residues corresponding to the hydrophobic patch (W26, F87 and I237) identified in AaL are highlighted in pink and the amino acids involved the metal coordination (including the characteristic HXHXDH motif) are designated by a red star. The position highlighted in green (Y223 in AaL) corresponds to a tyrosine residue possibly involved in the catalytic mechanism. The amino acids in red are involved in the dimerization of AaL.

The AaL protein sequence was aligned with sequences of MLLs with known structures using MUSCLE program^38^ (Fig. 2). AaL is closer to AiiB (43% sequence identity), whereas it shares only 21 and 27% sequence identity with AidC and AiiA, respectively. All four enzymes possess the characteristic *HXHXDH* motif, as well as the two other residues (H and D) involved in the metals cations coordination. Other residues lining the active site do not show significant conservation, with the exceptions of Y223 (Fig. 2). Indeed, Y223 is conserved in all the known MLLs, with the exception of AidC^35^ where it is substituted by a His. Interestingly, this residue is also conserved in PLLs^13,21^, and has been proposed to be implicated in the catalytic mechanism^31,39^.

### AaL is a proficient AHLs and lipophilic lactone hydrolase

AaL shows a wide activity spectrum against AHLs, as it hydrolyzes with similar, high catalytic proficiencies (up to 10^6^ M^−1^ s^−1^) AHLs with short, medium or long acyl chains (Table 2). This behavior has been previously observed for MomL^29^, AiiA^40^ and GcL^30^. Additionally, AaL is also able to degrade the γ- and δ-lactones with high proficiency. While this feature was not reported for MLLs, PLL-As, but not PLL-Bs, are also known to hydrolyze both AHLs and lipophilic lactones^21,23,36,41,42^. Interestingly, γ-butyrolactone derivatives are known autoinducers for *Streptomyces sp*., potentially suggesting a biological function to this activity^43^. Remarkably, the K_M_ values for AaL are low, as compared to most MLLs, and ranges between 9–83 μM with the exception of γ-butyrolactone (Fig. S3).

**Table 2:** Enzymatic characterization of the AaL enzyme. Related chemical structures of substrates are presented in Table S1.

Substrates	k_cat_ (s ^−1^)	K_M_ (μM)	k_cat_/K_M_(M ^−1^ s ^−1^)
C4- AHL (l)*	12.6 ± 0.8	9.4 ± 2.5	(1.3 ± 0.4) × 10^6^
C6- AHL (l)*	14.0 ± 0.4	82.7 ± 11.0	(1.7 ± 0.2) × 10^5^
C10- AHL (l)*	5.2 ± 0.3	57.2 ± 17.4	(9.2 ± 2.8) × 10^4^
3-Oxo-C12-AHL (l) *	4.9 ± 0.3	17.4 ± 4.8	(2.8 ± 0.8) × 10^5^
γ-butyrolactone	23.5 ± 1.7	171.0 ± 36.1	(1.4 ± 0.3) × 10^5^
γ-heptanolide	7.0 ± 0.3	36.9 ± 8.9	(1.9 ± 0.4) × 10^4^
δ-valerolactone	8.4 ± 0.4	18.7 ± 0.4	(4.5 ± 0.9) × 10^5^
δ-decalactone	8.4 ± 0.6	18.5 ± 0.4	(4.5 ± 1.0) × 10^5^
Paraoxon-ethyl	ND	ND	22.9 ± 1.4

Kinetics were measured as quadruplicates, and standard deviation values are given for each parameter. The kinetics were performed in *activity buffer* (2.5 mM Bicine pH 8.3, 150 mM NaCl, 0.2 mM cresol purple, 0.5**%** DMSO and with 0.2 mM CoCl_2_). ND corresponds to not determined values because of too low catalytic rate. *Data from^34^.

Additionally, we report that AaL exhibits some promiscuous, weak organophosphorus hydrolysis activity with a k_cat_/K_M_ value of 22.9 M^−1^ s^−1^ using paraoxon as a substrate. This trait has not been reported for other MLLs, but has been repeatedly described for PLLs. Some PLLs members were in fact identified by virtue of their phosphotriesterase activity^21,44,45^. It has been hypothesized that lactonases, owing to their ability to hydrolyze organophosphorus compounds, are the progenitors of optimized phosphotriesterases^46^.

### AaL is a quorum quenching lactonase

**Figure 3:**
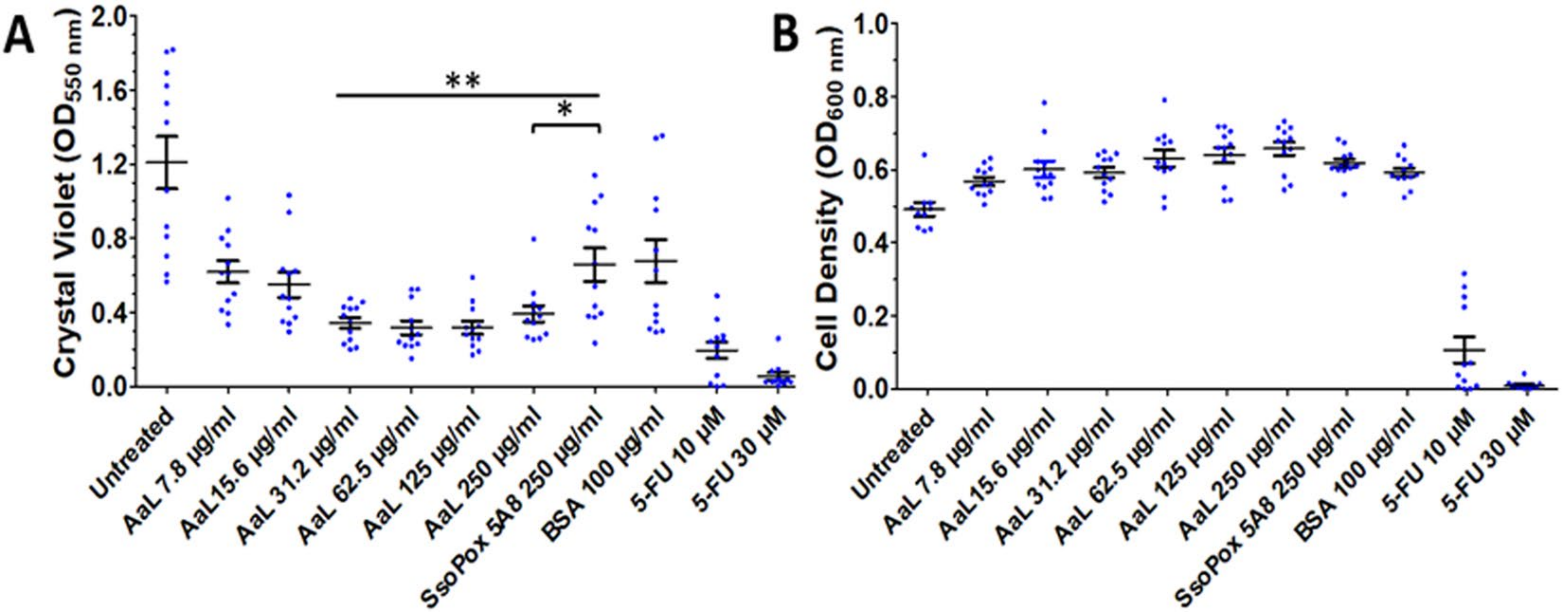
Effect of AaL on biofilm formation by Acinetobacter baumannii (**A**) and cell density (**B**) Biofilm was quantified using crystal violet staining and amount of biofilm is expressed as absorbance (550 nm). The cell density is a measure of cell growth in suspension measured as absorbance at 600 nm. The inactive mutant of the lactonase SsoPox (5A8) containing 10 mutations (V27G/P67Q/L72C/Y97S/Y99A/T177D/R223L/L226Q/L228M/W263H) and Bovine Serum Albumin (BSA) were used as negative controls while quorum sensing inhibitor (QSI) 5-fluoro-uracil has been used as positive control. Data shown are average of three independent experiments with 4 technical replicates each (n = 12), error bars represent the standard deviation. Student t-test results are shown (A) between data for biofilm formed with different doses of AaL and data for biofilm formed in presence of the inactive lactonase 5A8.

*Acinetobacter baumannii* is a human pathogen that is known to produce and utilize acyl homoserine lactone, including C12-AHL^47^. Quorum quenching enzymes, and in particular lactonases, were recently demonstrated to inhibit its biofilm formation^6,48^, and even disrupt existing biofilms^49^. Here we performed a biofilm inhibition dose-response experiment (Fig. 3). This experiment shows that AaL can inhibit the formation of biofilm, up to >4-fold as referred to untreated cultures, and >2.2-fold compared to an inactive lactone control (inactive mutant SsoPox 5A8; carrying the mutations V27G/P67Q/L72C/Y97S/Y99A/T177D/R223L/L226Q/L228M/W263H, obtained previously^50^). Interestingly, and as previously observed for the lactonase SsoPox^51,52^, AaL has no negative effects on growth, and exhibits similar growth levels as the controls performed with Bovine Serum Albumine (BSA) and 5A8. Control made with the inactive lactonase variant 5A8 suggests that the observed biofilm inhibition depends on catalytic activity. This observation contrasts with the use of the previously reported Quorum Sensing Inhibitor (QSI) 5-FU^53^ that inhibits both growth and biofilm formation.

### Crystal structure of AaL

**Figure 4:**
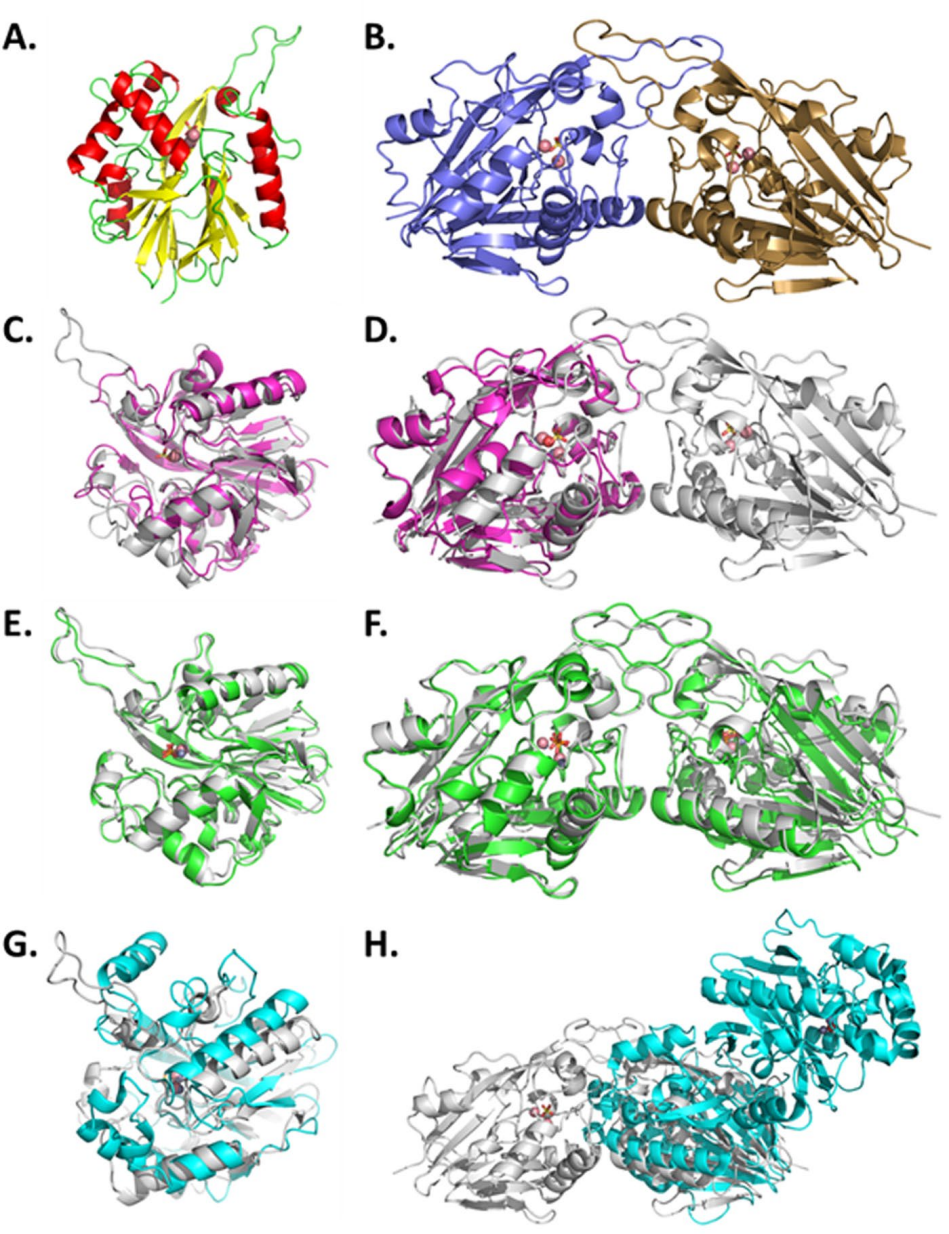
Structures of AaL and comparison to other known structures of MLLs. (**A**) AaL monomer shows a αβ/βα fold that contains two metal cations (pink spheres) and a bound phosphate anion (orange sticks). The α-helices, β-sheets and loops are represented in red, yellow and green, respectively. (**B**) AaL dimer. Each monomer (in blue and gold) contains an active site with two cobalt cations (pink spheres) bound to a phosphate anion (orange sticks). (**C**) Comparison of AaL (grey) and AiiA (pink) monomers. (**D**) Superposition of AaL dimer (grey) with the AiiA (pink) monomer. (**E**) Superposition of the monomers AaL (grey) and AiiB (green). (**F**) Comparison of the dimers of AaL (grey) and AiiB (green). The two enzymes show a similar dimerization mode. (**G**) Superposition of the monomer of AaL (grey) and AidC (cyan). (**H**) Superposition of AaL (grey) and AidC (cyan) dimers reveals their use of a different dimerization interface.

Structures of AaL were solved in C2 space group and in two different unit cells parameters (Table 3). The monomer of AaL is roughly globular with overall dimension of 60 Å × 52 Å × 43 Å approximately, and shows a long protruding loop (Fig. 4A). This loop is involved in homodimerization (Fig. 4B). The homodimer measures approximately 94 Å × 52 Å × 43 Å. As expected, AaL exhibits a αβ/βα sandwich fold, typical to the metallo-β-lactamase superfamily, and is similar to that of others MLLs such as AiiB or AiiA^26,54^. The overall structure is similar to the structure of AiiB (2R2D) from *Agrobacterium tumefaciens* (43% sequence identity). The root-mean-square deviation (r.m.s.d) for α-carbon atoms (over 273 atoms) is 0.95 Å.

**Table 3:** Data collection and refinement statistics of AaL structures.

Structure	AaL bound to phosphate	AaL bound to glycerol	AaL bound to C6 AHL
**DATA COLLECTION STATISTICS**			
PDB ID	6CGY	6CH0	6CGZ
Diffraction source	APS Argonne 23ID-B	APS Argonne 23ID-D	APS Argonne 23ID-D
Wavelength (Å)	1.03323	1.03321	1.03321
Detector	EIGER-16M	PILATUS3–6M	PILATUS3–6M
Rotation range per image (°)	0.2	0.2	0.3
Total rotation range (°)	200	240	230
Space group	C2	C2	C2
Unit-cell parameters (Ǻ)	a = 111.72	a = 110.84,	a = 145.94
	b = 114.74	b = 113.50	b = 88.69
	c = 79.97;	c = 79.10;	c = 97.68;
	α = 90.000	α = 90.000	α = 90.000
	β = 109.778	β = 108.907	β = 128.240
	γ = 90.000	γ = 90.000	γ = 90.000
Resolution range (Ǻ) (last bin)	1.65 (1.65–1.75)	2.15 (2.15–2.25)	1.8 (1.8–1.9)
Total N° of reflections (last bin)	431338 (70385)	217409 (28557)	378841 (57949)
N° of unique reflections (last bin)	111796 (17858)	50025 (6321)	90111 (13463)
Completeness (%) (last bin)	98.2 (97.2)	99.3 (99.0)	99.2 (99.3)
Redundancy	3.86 (3.94)	4.35 (4.52)	4.20 (4.30)
〈*I*/σ(*I*)〉	22.08 (3.16)	11.71 (2.73)	18.27 (2.71)
*R*_sym_(%)	3.1 (51.1)	7.0 (61.5)	4.8 (52.2)
CC(1/2)	100 (84.9)	99.8 (85.5)	99.9 (81.6)
**REFINEMENT STATISTICS**			
Rfree/Rwork (%)	19.73/17.17	22.92/18.75	19.24/16.49
N° of total model atoms	7311	6979	7602
Ramachandran favored (%)	94.17	95.09	94.72
Generously allowed rotamers (%)	3.44	2.97	3.52
Ramachandran outliers (%)	2.38	1.94	1.76
Rmsd from ideal			
Bond lenghts (Ǻ)	0.030	0.017	0.023
Bond angles (°)	2.423	1.753	2.082

**Figure 5:**
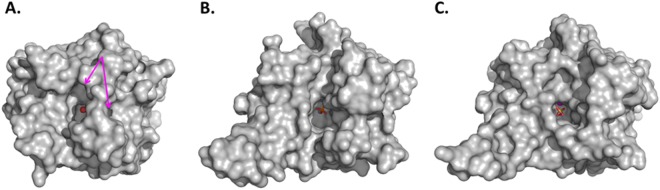
Active site accessibility in three different MLLs. (**A**) AiiA (PDB: 2A7M) active site cavity appears divided in two parts by two residues (F68 and E135), indicated by pink arrows. (**B**) AiiB (PDB: 2R2D) active site cavity. AiiB is bound to a phosphate molecule shown as orange sticks. (**C**) AaL (PDB: 6CGY) active site is bound to a phosphate molecule shown as orange sticks. Active site metal cations are shown as red spheres.

AaL shows highly significant differences with AiiA with a r.m.s.d for α-carbon atoms (overs 181 atoms) at 1.22 Ǻ. One of the most important difference between the structure of AiiA and AaL resides in an external loop involved in the dimerization. This external loop is produce by an insertion of nine amino acids between the residues P33 and A41 (Fig. 2). Another noticeable difference between AiiA and AaL resides in the access to the active site. In fact, AaL active site appears much more accessible than AiiA’s active site, which is partially obstructed by two residues (E135 and F68 in AiiA) (Fig. 5A,C).

AaL and AidC shares 21% of sequence identity, and despite exhibiting a similar topology, their structures show major differences. The most important differences are localized in the active site area, with a different orientation of the active site crevice^28^, as well as a different homodimerization surface^28^ (Fig. 4G,H).

The finding that AaL, isolated from a thermoacidophilic bacteria, and contrary to AiiA^14^, is organized as a homodimer, is consistent with previous work on thermophilic proteins, highlighting a trend for higher levels of oligomerization in these proteins^55^. The dimer is characterized by a strong interaction of the protruding loop (P33 to A41) from both monomers (Fig. 4B). The dimer interface involves 32 residues in each monomer. The interface is mostly hydrophobic, and involves only 14 hydrogen bonds. The contacting area is 1125.7 Å^2^, a similar value to other dimeric MLL structures such as AiiB and AidC, 1089.1 Å^2^ and 1015.4 Å^2^, respectively.

### Active site of AaL

AaL possesses a bi-metallic active site center. The bound metal cations nature in the MLLs was previously attributed to two zinc cations in AiiA^26^ as well as AiiB^54^. Metal cations are bound via five histidine residues (118, 120, 123, 198 and 266), and two aspartic acid residues (122 and 220). Anomalous X-ray scattering data collected at a higher energy than the Co-Kedge (7.7089 keV) shows that the active site may be occupied by cobalt cations, but not by other common metal cations identified in similar enzymes such as zinc (Zn-K edge is 9.6586 KeV) or nickel (Ni-K edge is 8.3328)^21,56^ (Fig. S2). This result contrasts with known enzymes from the MLL family that were described to possess two zinc cations in their active site^35,39,54^ (Fig. S2), but is consistent with the fact that cobalt cations were added to the protein production steps. Therefore, cobalt was modelled in the active site of AaL.

The crystal structure of AaL reveals that its active site is hydrophobic. Besides tyrosine 223, a residue conserved in lactonases and potentially involved in the catalytic mechanism^31,32,54,57^, the active site cavity is decorated by the side chains of three methionine residues (20, 22 and 86), a tryptophan residue (26), a phenylalanine residue (87), an isoleucine residue (237), a leucine residue (121) and an alanine residue (157). The hydrophobic character of the cleft is reinforced by W200 and Y239 that might interact with the side chains of very long chain AHLs.

#### Comparison with AiiA

**Figure 6:**
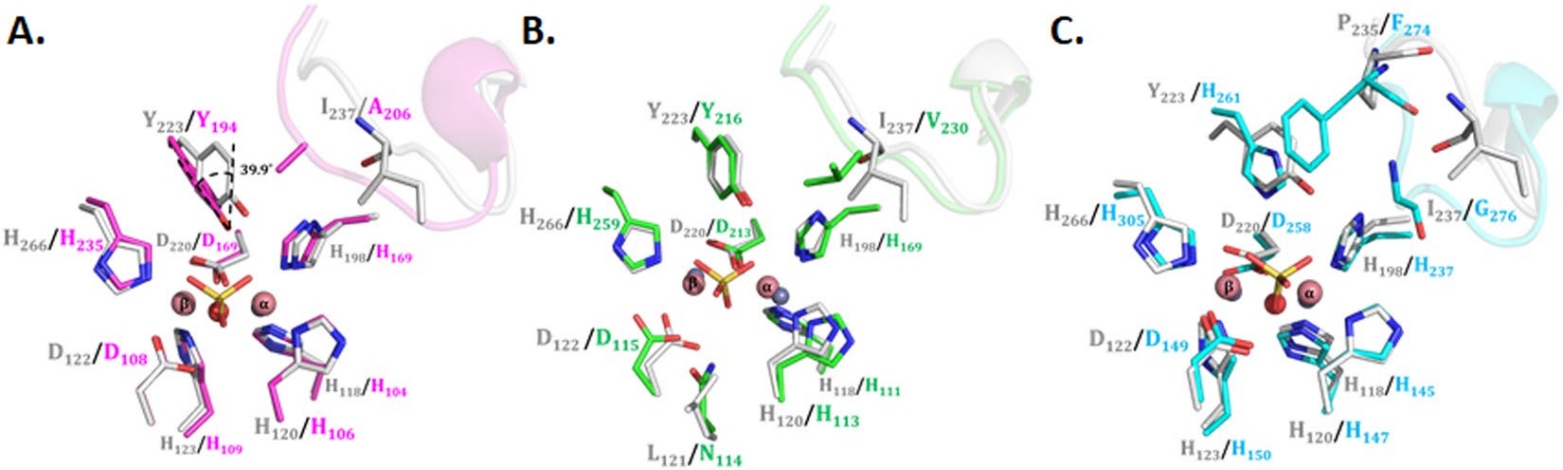
Comparison of AaL active site with AiiA, AiiB, and AidC. (**A**) Superposition of AaL (in grey sticks) and AiiA (in pink sticks) highlights a reorientation of Y223/Y194 and of the active site loop (237-loop in AaL). (**B**) Superposition of AaL (grey sticks) and AiiB (green sticks) reveals a different metal coordination geometry (AaL cations are shown as pink spheres, AiiB cations as grey spheres) and the substitution I237/V230. (**C**) Superposition of AaL (grey sticks) and AidC (cyan sticks) active sites. Active site residues occupies similar configurations, with the exception of the Y223/H261 substitution, and residues on loops (P235/F274 and I237/G276). The phosphate-bound structure of AaL was used in these figures. Metal cations (pink spheres for AaL, grey spheres for other enzymes) and the bridging water molecule are shown as well as the bound anions shown as sticks.

Both enzymes differ significantly in their active site accessibility. While AiiA has a relatively narrow active site entrance, AaL has a much more accessible active site center (Fig. 5A,C). This is due to different conformations of loops decorating the active sites of both enzymes, mainly caused by an insertion of seven amino acids (Y149 to E155) in AaL as compared to AiiA (Figs 2 and S4). Another difference relates to a loop surrounding the active site and containing I237: AaL exhibit a 2-residues insertion next the active site (P235 and G236) as compared to AiiA. This insertion affects the position of I237 which is lining the active site (Fig. 6A), and may alter the relative binding of lactones onto these two enzymes (Fig. S5). Lastly, whereas the key active site amino acids are well superposed (Fig. 6A), Y223 (in AaL) and the equivalent residue in AiiA (Y194) adopt a twisted orientation (39.9°). Given the possible implication of this residue for catalysis^31,32,54,57^, this may have implications on the role of this residue in both enzymes (Fig. 6A).

#### Comparison with AiiB

While AaL and AiiB share very similar structures, some differences can be seen while comparing their respective active site (Fig. 6B). First, W200 in AaL (S in AiiB), a bulky residue, affects the position of the neighboring Y158 (Y151 in AiiB) and H120 (H113 in AiiB) (Fig. S6). As a result, the metal coordinating residue H120 as well as the metal cations adopt different positions in the two enzymes. In fact, metal coordination is affected and the distance between both metal cations varies greatly between the two enzymes (3.5 Å and 4.2 Å in AaL and AiiB, respectively, for the two phosphate-bound structures) (Fig. 6B). Concurrently, other metal coordinating residues adopt slightly different conformations such as H118 and D122, respectively H111 and D115 in AiiB (Fig. 6B).

Other minor differences can be observed. In the I237-containing loop (237-loop), residue I237 (in AaL) corresponds to V230 in AiiB (Fig. 6B). Some other residues lining the active site also differ. Indeed, as shown by the sequence alignment (Fig. 2), very few residues putatively involved in substrate binding are conserved. Noteworthy, most substitutions between AaL and AiiB (F19_AiiB_/W26_AaL_; A80_AiiB_/F87_AaL_; V230_AiiB_/I237_AaL_; N114_AiiB_/L121_AaL_; V15_AiiB_/M22_AaL_) suggest a more hydrophobic active site pocket for AaL. In particular, residues W26, F87 and I237 in AaL create a unique hydrophobic patch not present in other MLLs structures.

#### Comparison with AidC

The entrance of the active site binuclear center in AidC is located on a different face of the enzyme, as compared with AiiA, AiiB, and AaL. AidC and AaL share a similar metal coordination (Fig. 6C), but due to the previously described reorganization of the AidC binding cleft^28^, their binding cleft are different. Noteworthy the conserved residue Y223 in MLLs, as well as in PLLs^13^, possibly involved in the catalytic mechanism, is replaced by an histidine residue in AidC^35^ (Fig. 6C).

### Structure of AaL bound to a phosphate anion

**Figure 7:**
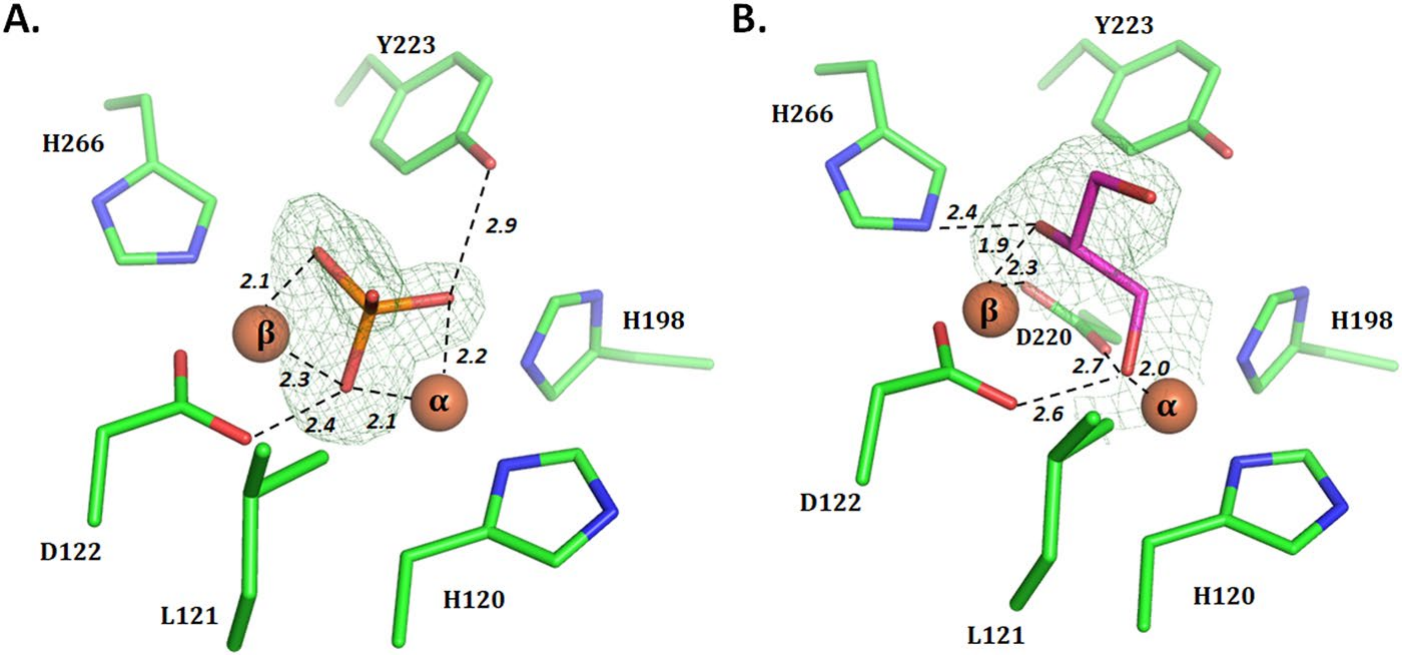
AaL active site in structures bound to phosphate and to glycerol. (**A**) Structure of AaL (green sticks) bound to phosphate (orange sticks) (PDB ID: 6CGY). 3 out of the 4 oxygen atoms of the phosphate molecule interact closely with the two metal cations α and β and as well as with D122 and Y223. (**B**) Structure of AaL (green sticks) bound to glycerol (pink sticks) (PDB ID: 6CHO). The glycerol molecule interacts with the bimetallic active site via two oxygen atoms. Fourier difference maps (F_o_-F_c_) shown in green mesh are calculated by omitting the ligand from the model, and contoured at 5.0 σ (**A**) and 2.5 σ (**B**) level.

Examination of one of the obtained AaL structure reveals the presence of a tetrahedral molecule in the active site, as clearly illustrated by the electronic density (Fig. 7A). Similarly to what was observed in the AiiB structure^54^, this density was attributed to a phosphate anion. In this structure, the α-metal is bridged with H118, H120, H198, D220 and two phosphate oxygen atoms, while the β-metal is bound to H123_,_ H266, D122, D220 and two oxygen atoms from the phosphate anion. With the bound phosphate, the bridging water molecule is not present in the metal coordination. The presence of this tetrahedral anion in the active site of lactonases might mimic the tetrahedral geometry of the lactone hydrolytic transition state^2^.

Interestingly, the phosphate-bound AaL and AiiB structures differ in the way they coordinate the anion. In particular, in AiiB, N114 strongly interacts with one of the oxygen atom of the bound phosphate molecule (Fig. S7), demonstrating its ideal position to create polar interactions with molecules bound to the bi-metallic active site center, and thereby including lactones. However, in the case of AaL, the corresponding residue, a leucine, does not interact with the bound anion.

### Structure of AaL bound to a glycerol molecule

AaL was also crystallized bound to a glycerol molecule. Glycerol may originate from the cryoprotectant solution that contains glycerol^34^. Similar cryoprotectant molecule (ethylene glycol) was previously observed to bind to bimetallic enzyme active sites in PTE^58^. The molecule interact through two of its alcohol groups with the metal cations and with D122. In fact, an alcohol group is bridged with the α-metal, D122 and D220 at 2.0, 2.6 and 2.7 Å respectively and the second alcohol interacts with the β-metal (1.9 Å) (Fig. 7B). Surprisingly, whereas the metal coordination is very similar to other lactonases from the MLL family (AiiA, AiiB, AidC) and the PLL family (SsoPox, SisLac, VmoLac), and the good resolution of the structure (2.15 Å), the putative metal cation-bridging water molecule is not visible in the electronic density maps. It is however difficult to conclude whether this molecule is present or not, since the observation of this water molecule can be difficult because of the strong density peaks corresponding to both metal cations, and the conserved coordination suggests that the conserved bridging water is present in AaL.

When compared to the AaL structure bound to phosphate, both structures align very well, with the exception of the 237-loop (Fig. S8A). Additionally, the metal coordination is slightly different in the two structures. Indeed, the distance between the two metal cations is bigger in the glycerol bound structure, as compared to the phosphate bound one (4.0 Å and 3.5 Å, respectively). This lead to a shift in the position of the residues H120, H123, H266 and D220 which coordinates the metals (Fig. S8B).

### Complex with C6-AHL

**Figure 8:**
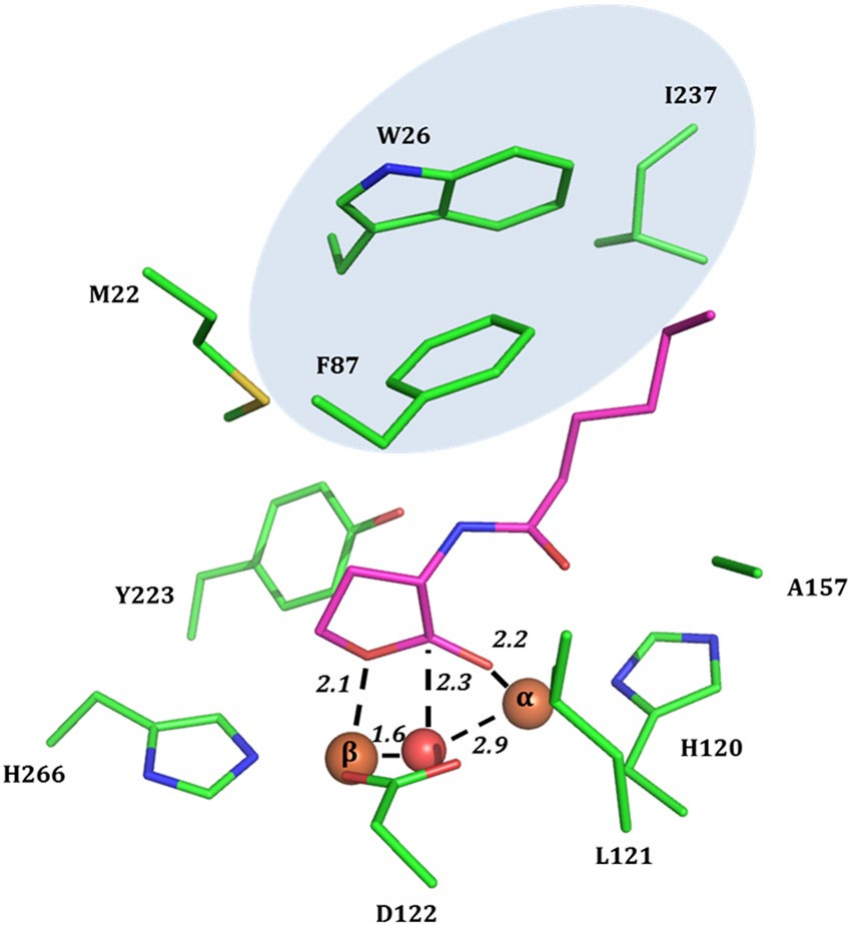
AaL complex with a C6-AHL. Active site residues (green sticks) are shown and interactions between the bound C6-AHL (purple sticks), metal cations, and the bridging water molecule are highlighted as black dashes and distances are indicated in Ångstroms. The hydrophobic patch accommodating the AHL acyl chain is highlighted with a light blue background.

After soaking AaL crystals in the cryoprotectant solution supplemented with 20 mM of C6-AHL for 10 min, the structure of AaL bound to C6-AHL could be solved. C6-AHL is bound within the active site (Fig. 8) with a modelled 0.7 occupancy (Fig. S9). As a result of soaking, the crystals belong to the same space group than the two other structures (C2), but the unit cell parameters are different (Table 3). This structure is overall very similar to the other structures of AaL, including in the metal coordination sphere. Metal cations are distant by 4.0 Å. A slight change of conformation of I237 is observable in the C6-AHL-bound structure as compared to the phosphate-bound structure (Fig. S10).

The lactone ring interacts with the bi-metallic active site (Fig. 8). The carbonyl oxygen of the ring is bound to the α-cobalt (2.2 Å) and the esteric oxygen with the β-cobalt (2.1 Å). The catalytic water is at 2.3 Å of the nucleophilic carbon that it will attack to degrade the substrate. This binding configuration is compatible with the previously observed binding mode of a hydrolytic product in AiiA^54^, but significant differences are observable (Fig. S11). Notably, the bridging water molecule is interacting more closely with one of the metal cation (β-metal) than with the other metal (α-metal), as reported for PLLs^21^. This configuration is compatible with the proposed mechanism utilizing the activating bridging catalytic water as the attacking nucleophile to open up the lactone ring^26^.

The accommodation of the N-alkyl chain of the AHL is unique: whereas AiiA utilizes a wide and shallow crevice, where longer AHL residues can be stabilized by a phenylalanine clamp^54^, the binding cleft in AaL is different. The binding cleft is much wider than in AiiA’s structure, very hydrophobic, revealing an additional hydrophobic cluster, or patch (Fig. 8). The structure shows that the acyl chain interacts with the hydrophobic patch formed by W26, F87 and I237 that has no equivalent in other MLLs (Fig. S12), and this results in a different orientation of the alkyl chains in AaL, as compared to AiiA (Fig. S11). The presence of unique hydrophobic patch may contribute to the observed lower K_M_ values of AaL. Additionally, the bound C6-AHL also interacts with M20, M22, M86, Y223, L121 and A157. Remarkably, as compared to the phosphate-bound structure, the 237-loop adopts a slight reorientation upon C6-AHL binding, including a reorientation of I237 side chain (Fig. S10). The importance of the interaction of I237 with the acyl chain of C6-AHL is further evidenced by the decreased of its mobility in the C6-AHL-bound structure, as compared to the phosphate-bound structure, as evidenced by normalized thermal motion factors (B-factors) (Fig. S13).

## Conclusion

The quorum quenching lactonase AaL from the thermoacidophilic bacterium *Alicyclobacillus acidoterrestris* exhibits a very broad substrate range, being capable of hydrolyzing short and long chain AHLs with high proficiencies. This broad substrate specificity seems common to most of the MLL lactonases identified thus far, including GcL^30^, MomL^29^, AidC^28^ or AiiA^40^. The ability of AaL to hydrolyze AHLs results in its capacity to inhibit the biofilm formation of *Acinetobacter baumannii*. Additionally, AaL exhibits high catalytic proficiency against δ-lactones and γ-lactones. This is noteworthy, because some γ-lactones are used as QS molecules in *Streptomyces* and *Rhodococcus*^24,25^.

Furthermore, its promiscuous ability to degrade the phosphotriester paraoxon resonates with previous studies suggesting an evolutionary link between phosphotriesterases (PTEs) and lactonases^13,46^. Indeed, lactonases might be the progenitors of modern PTEs^46^, which are insecticide-degrading enzymes hypothesized to have emerged in the last 70 years to specifically degrade man-made organophosphorus insecticides. Remarkably, for all three main lactonase families, namely the PLLs, the PONs, and the MLLs, closely related PTE representatives could be identified and characterized^13,17,59–61^. The weak, promiscuous paraoxonase activity of AaL may confirm the previously observed catalytic relationships between PTEs and lactonases^13,17,46^.

Lastly, the unusually low K_M_ values of AaL correlates with the presence of a hydrophobic patch in the vicinity of the active site that is unique to AaL structure. Structural analysis of the structure bound to a C6-AHL molecule allows for the identification of the residues interacting with the acyl chain. In particular, a residue within this hydrophobic patch, I237, adopts slightly different conformations upon the binding of the C6-AHL molecule, suggesting a potential role in the AHL accommodation. The use of lactonase with low K_M_ values may be of particular interest to increase their quorum quenching abilities. Indeed, the majorities of quorum quenching enzymes identified so far have high apparent dissociation constant values (100–1000 µM). These values contrast with the reported activation threshold of QS for numerous bacteria, in the range of ~5 nM^62–64^. Future investigations will reveal if the use of lactonases with lower K_M_ values result in stronger quenching, and AaL will serve as an example to understand how lactonases can achieve low apparent dissociation constant values.

## Methods

### Sequence alignment

The FASTA protein sequence of AaL was blasted against the non-redundant protein database to collect the sequences of previously characterized lactonases. The protein sequence of AaL (WP_021296945.1) contains 282 amino acids and has a molecular weight of 32.0 kDa. The alignment of AaL and other AiiA-like lactonase representative was performed in MEGA^65^ by using the software MUSCLE^38^

### Cloning, expression and purification of the protein AaL

The gene encoding for AaL has been optimized for heterologous expression in *Escherichia coli* and synthesized by Genscript (Piscataway Township, New Jersey, USA). To performed production, AaL gene was built by the addition of a N-terminal affinity strep tag (WSHPQFEK) followed by a TEV cleavage site (ENLYFQS). AaL production was performed in *E*. *coli* BL21 (DE3)-pGro7/ GroEL strain (Takara). The production was effected by using ZYP autoinducer media at 37 °C until cells reached the exponential growth phase (OD_600nm_). Then, the cultures were transferred at 18 °C overnight and 0.2 mM CoCl_2_ were added, as well as 0.2% of L-arabinose to induce the chaperon. The use of cobalt during induction and in subsequent purification steps was previously used for the purification of similar lactonases and was associated to increased induction and activity levels^44^. The cells were collected by centrifugation at 4 °C (4400 g during 15 minutes) and resuspend in lysis buffer (50 mM HEPES pH 8.0, 150 mM NaCl, 0.2 mM CoCl_2_, 0.25 mg.ml^−1^ lysozyme, 0.1 mM phenylmethylsulfonyl (PMSF)). After 30 minutes in ice, cells were lysed by using a sonicator device (Q700 Sonicator Qsonica, USA) during 3 times 30 seconds (1 second pulse-on; 2 seconds pulse-off). The lysate was finally loaded on a Strep Trapp HP chromatography column (GE Healthcare) at room temperature. StrepTag encoding in AaL gene were thereafter cleaved by using TEV protease (Tobacco Etch Virus protease) at a ratio of 1/20 w/w, during 20 hours at 4 °C. After cleavage, protein sample were concentrated, filtered at 0.22 μm and loaded on a size exclusion column (Superdex 75 16/60, GE Healthcare). To identify and control the protein purity, samples were loaded on SDS-PAGE gel (Fig. S1).

### Kinetic measurements

Reaction rates of AaL enzyme were monitored using a microplate reader (Synergy HTX, BioTek, USA) and the software Gen5.1 in 96-well plates of 5.8 mm path lengh for a 200 μl reaction at room temperature. Measurements were performed in at least triplicates. The catalytic parameters were obtained by fitting the data to the Michaelis-Menten equation with the *Graph-Pad Prism 5* software. In case were *V*_*max*_ could not be reached, the catalytic efficiency was obtained by fitting the linear part of Michaelis-Menten plot to a linear regression.

*Paraoxon assay*. The ethyl-paraoxon hydrolysis was performed in *PTE buffer* (50 mM HEPES pH 8.0, 150 mM NaCl, 0.2 mM CoCl_2_). Catalytic parameters were evaluated using 1.45 µM of enzyme and paraoxon concentrations ranging from 0 to 6 mM. The assay were performed by measuring the production of paranitrophenolate anions (ε_405nm_ = 17 000 M^−1^ cm^−1^) as previously described^23,42^ (Fig. 1).

*Lactonase assay*. The hydrolysis of lactones, *via* the opening of the lactone ring, increases the acidity of the solution by generating a proton. A pH indicator, the cresol purple (pKa 8.3 at 25 °C) was used to monitor the acidification of the medium induced by the hydrolysis of the lactone ring as previously described^30,34^. The time course of the hydrolysis of the lactones was recorded at 577 nm in the *activity buffer* (2.5 mM Bicine pH 8.3, 150 mM NaCl, 0.2 mM cresol purple, 0.5% DMSO and with 0.2 mM CoCl_2_). The assays were performed using AHLs as substrates: C4-AHL, C6-AHL, C10-AHL, 3-oxo-C12-AHL and using other lactones: γ-butyrolactone, γ-heptanolide, δ-valerolactone, δ-decalactone as substrates (Table S1). The concentration range for the different lactones varied between 0.001 mM and 1.5 mM. The enzyme concentrations used for kinetic experiments were adjusted as a function of observed velocities to optimize the quality of the acquired data sets. For each tested substrate, a specific concentration of enzyme, between 1.45 µM and 0.73 µM, was used. The kinetics parameters of AaL against AHLs C4-AHL, C6-AHL, C10-AHL, 3-oxo-C12-AHL were previously published^34^.

### Data collection, structure resolution and refinement

Crystals of AaL bound to phosphate and bound to glycerol were obtained in conditions previously reported^34^. Crystals bound to C6-AHL were obtained after soaking the crystals in a cryoprotectant solution supplemented with 20 mM C6-AHL for 10 min. AaL structure was collected and obtained by X-ray diffraction intensities at APS-Argonne on the beamline 23ID-B (Lemont, Illinois, United states) using an EIGER detector. The collection were performed at 1.03323 Ǻ wavelength and 1000 images were collected (0.2 s exposure; 0.2° step). The *XDS software package*^66^ was used to integrate and scale X-ray diffraction data. The closest known structure to AaL, AiiB from *Agrobacterium tumefaciens* (43% sequence identity)^54^ (PDB: 2R2D) was used as a template for molecular replacement procedure using MOLREP^67^. Buccaneer software^68^ was used to automatically build a partially model of AaL that was subsequently improved manually using the software *Coot*^69^. Refinement cycles were performed using REFMAC^70^ to improve the structural model and the final refinement statistics are available in Table 3.

### Anomalous X-ray scattering data

In order to determine the chemical nature of the two active-site metals, an anomalous data set was collected. Because the buffers used during the purification step contained salts, and because cobalt cations are known to be present in active sites of lactonases^21^, we collected a 2.4 Å resolution set at higher energy (7.715 keV) than the Co-K absorption edge (7.7089 KeV). (Table S2 and Fig. S2)

### Structure analysis

The crystals structures of AaL (PDB 6CGY), AiiA (PDB 2A7M and 3DHB), AiiB (PDB 2R2D) and AidC (PDB 4ZO2) were compared. PyMOL software was used to analyze, compare and illustrate our data^71^. The dimer interface surface together with the number of hydrogen bonds and salt bridges were computed using PISA32 server and default parameters^72,73^. The determination of the root mean square deviations (r.m.s.d.) were calculated on α -carbon using the align command under the Swiss-PdbViewer^74^.

### Biofilm inhibition assay

The bacterial strain *Acinetobacter baumannii* ATCC^®^ 19606^TM^ was used in this study. The bacteria were grown in nutrient broth (NB) medium (Fisher Scientific) as instructed by ATCC. The biofilm assays were carried out in MOPS minimal medium made according to Neidhardt *et al*. protocol^75^ (50 mM MOPS, 4 mM Tricine, 50 mM NaCl, 1 mM K_2_HSO_4_, 50 mM MgCl_2_, 10 mM CaCl_2_, 0.3 mM (NH_4_)_6_Mo_7_O_24_, 40 mM H_3_BO_3_, 3 mM Co(OAc)_2_, 1 mM CuSO_4_, 8 mM MnSO_4_, 1 mM ZnSO_4_, 25 mM sodium glutamate 15 mM NH_4_Cl, 4 mM K_2_HPO_4,_ 5 µM FeSO_4_ [pH 7.0]).

A single colony of *A*. *baumannii* was inoculated into 5 ml of NB medium and grown at 37 °C, 200 rpm shaking for 6 hr as pre-culture. The pre-culture was diluted 1:1000 in MOPS minimal medium and inoculated into a sterile polystyrene 96-well round-bottom plate (Costar) for biofilm assay with different concentration of the lactonase. The enzyme concentrations were prepared by serial dilutions and added to the 96-well plate in triplicates. The plate was incubated at 37 °C with continuous agitation and biofilm was assayed 16 hr post inoculation by crystal violet staining. The crystal violet staining was carried out as reported by O’Toole (2011)^76^. Briefly, the unattached cells were removed by gently pipetting out into another plate and the cell density was measured by absorbance at 600 nm using a plate reader (Synergy HTX multi-mode reader, BioTek). The culture plate was then washed by gently immersing it in tub full of distilled water and gently shaking out the water and blotting the plate dry. The biofilm was allowed to dry at room temperature for half an hour and then 200 µl of 0.1% crystal violet solution was added to each well. The crystal violet was discarded and the wells were washed as mentioned earlier. The crystal violet was solubilized using 30% acetone. 200 µl from each well were transferred to a fresh flat-bottom 96-well plate (Fisherbrand, Fisher Scientific) and the absorbance was measured at 550 nm.

## Electronic supplementary material

Supplementary Information
