# Supplementary material for: Structural and Biochemical Characterization of AaL, a Quorum Quenching Lactonase with Unusual Kinetic Properties
Source: Sci Rep. 2018 Jul 26;8:11262. doi: 10.1038/s41598-018-28988-5 (PMC6062542; doi:10.1038/s41598-018-28988-5)
Supplement: Supplementary file 1 — Supplementary Information [file 41598_2018_28988_MOESM1_ESM.docx]

**Supplementary Data**

**Structural and Biochemical Characterization of AaL, a Quorum Quenching Lactonase with Unusual Kinetic Properties**

Celine Bergonzi^a^, Michael Schwab^a^, Tanushree Naik^a^, David Daudé^b^, Eric Chabrière^c^ and Mikael Elias^a^*

^a^ Biochemistry, Molecular Biology & Biophysics Dpt and BioTechnology Institute, University of Minnesota, Saint Paul, Minnesota, 55108, USA

^b^ Gene&GreenTK, 19-21 Boulevard Jean Moulin, 13005 Marseille, France

^c^  Aix Marseille Univ, IRD, APHM, MEPHI, IHU-Méditerranée Infection, Marseille, France

Correspondence email: Prof. Mikael Elias; Tel: +1-612 626 1915; Fax: +1 612 625 5780; mhelias@umn.edu

Table of Contents

Table S1: Chemical structures of substrates used in this study **3**

Table S2: Anomalous data collection statistics **4**

Figure S1: SDS–PAGE of the lactonase AaL **5**

Figure S2: Anomalous scattering discriminates between metals in the AaL active site **6**

Figure S3: Kinetics parameters of AaL **8**

Figure S4: Difference in the active site accessibility of AaL and AiiA **9**

Figure S5: Overlay of the active sites of AaL and AiiA **10**

Figure S6: Active site comparison between AaL and AiiB **11**

Figure S7: Overlay of the active sites of AaL, AiiA and AiiB **12**

Figure S8: Comparison of the bound to phosphate and bound to glycerol structures **13**

Figure S9: Electronic density for the C6-AHL complex structure **14**

Figure S10: Superposition of the phosphate-bound and C6-AHL bound AaL structures.**15**

Figure S11: Overlay of the C6AHL-bound AaL and AiiA structures **16**

Figure S12: Overlay of the active sites of AaL, AiiA and AiiB. **17**

Figure S13: Comparison of normalized thermal B-factors of AaL structures **18**

References **19**


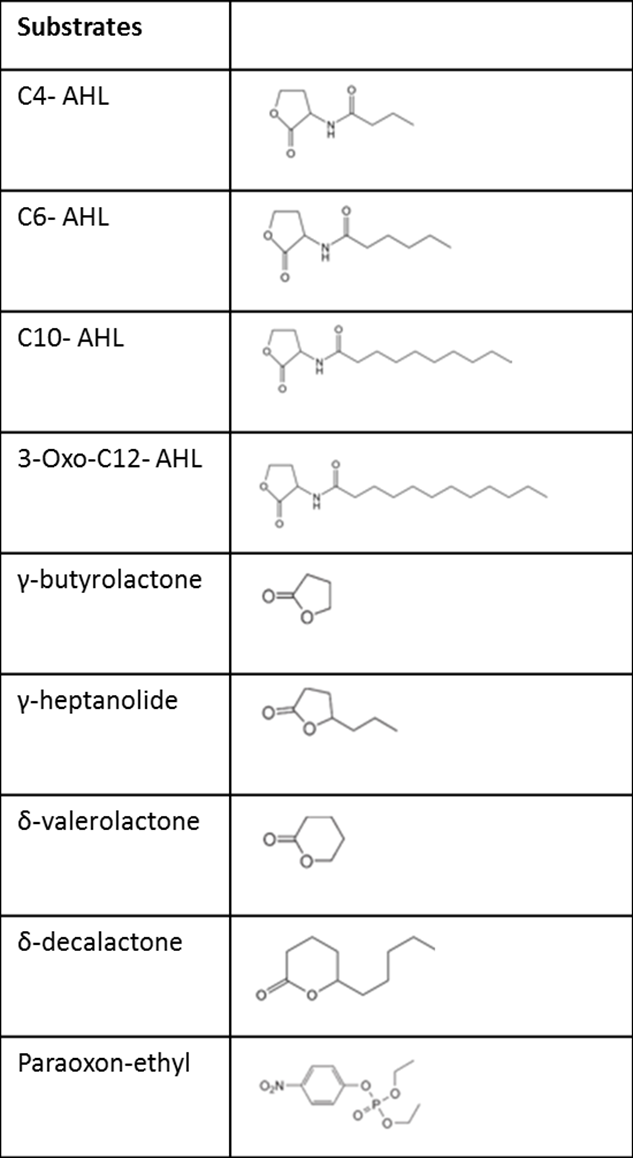


Table S1: Chemical structures of AHL, γ- and δ- lactones and ethyl-paraoxon used in this study.

| **DATA COLLECTION STATISTICS** | |
| --- | --- |
| **Diffraction source** | APS Argonne 23ID-B |
| **Wavelenght (Å)** | 1.607053 |
| **Temperature (K)** | 100 |
| **Detector** | EIGER-16M |
| **Crystal-detector distance (mm)** | 200.001 |
| **Rotation range per image (˚)** | 0.5 |
| **Exposure time per image (s)** | 0.2 |
| **Space group** | C2 |
| ***Unit cells parameters*** | *a= 111.6, b= 114.9, c= 79.7 ;*  *α=γ= 90.0, β= 109.6* |
| **Resolution range (Å)** | 2.5 (2.5-2.6) |
| **Total N˚ of reflections** | 217710 (24537) |
| **N˚ of unique reflections** | 62961 (6868) |
| **Completeness (%)** | 97.4 (96.1) |
| **Redundancy** | 3.46 (3.57) |
| **(*I/σ(I))*** | 25.16 (8.91) |
| **R*_meas_*(%).** | 4.0 (15.6) |

Table S2: Anomalous data collection statistics at a higher energy than Co-edge (7.7089 KeV).


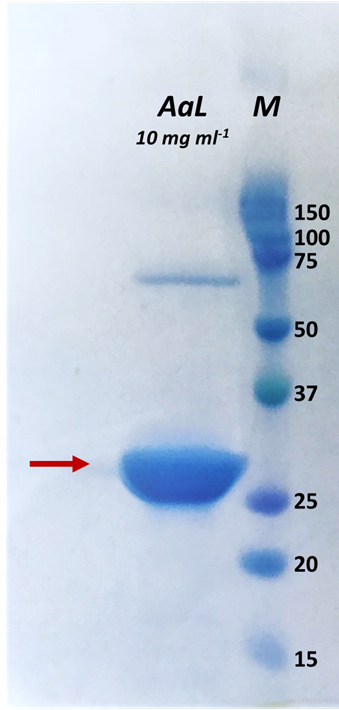


**Figure S1**: 12% SDS–PAGE of the purified AaL protein (full-length gel). The column M contains the molecular weight markers Precision Plus Protein Kaleidoscope Prestained Protein (Bio-Rad) labelled in kDa. The column AaL contains 10 µl of Protein concentrated at 10 mg ml^-1^. The red arrow is pointing the band corresponding to the protein AaL


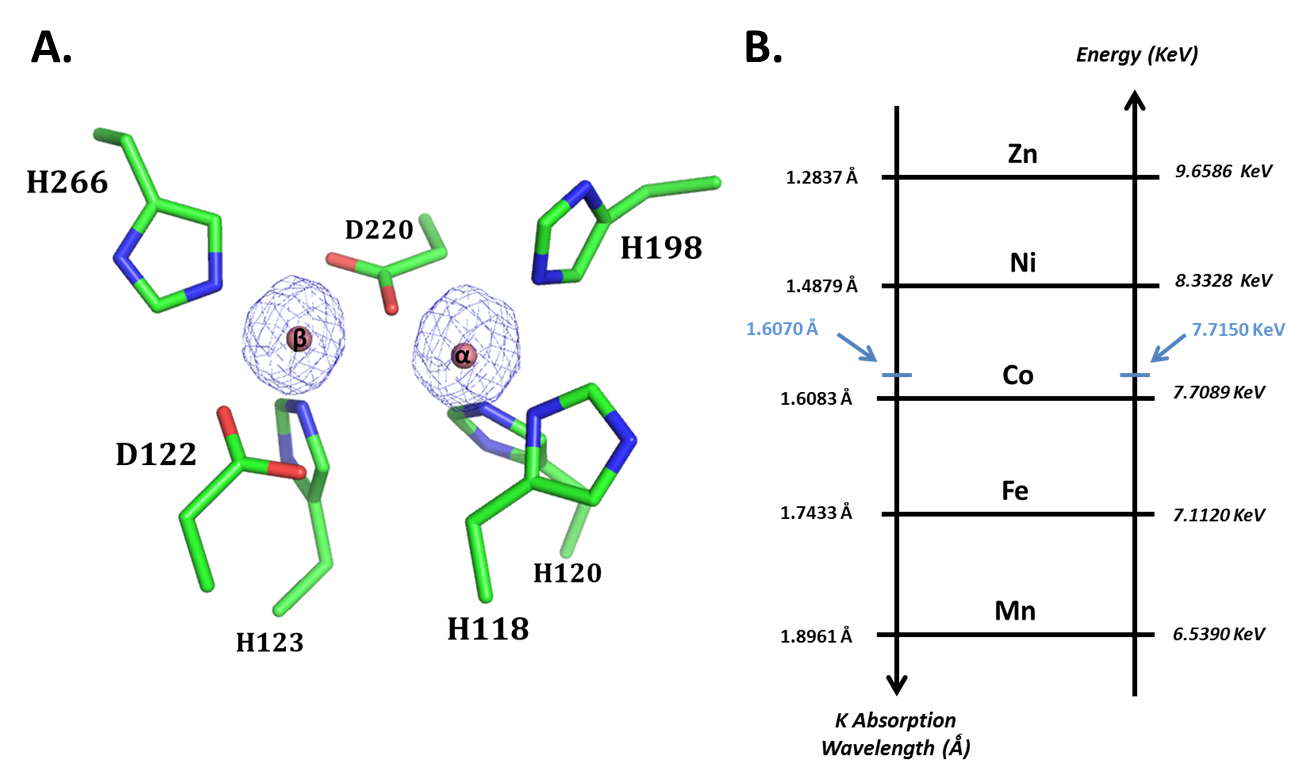


Figure S2: Anomalous scattering of bound metal cations in the active site of AaL. (A) Shown are final model (green sticks) and Bijvoet difference Fourier maps contoured at 5σ the map corresponding to data collected at 1.607053 Å. The maps were calculated using the full resolution range and the peaks height are 16.7σ and 16.9σ, for α-site and the β-site, respectfully. (B) Different edges of transition metals near the collected energy. Observation of two peaks at 1.607053 Å clearly demonstrates that α- and β-sites are not occupied by nickel or a zinc cations but by cobalt, iron or mangenese cations. Because cobalt was used during the production of this enzyme, cobalt cations were modelled in the structure of AaL.

**AaL is possibly a cobalt-containing metalloenzyme**. The chemical nature of metalloenzymes can be ambiguous^1^, and active sites metals were shown to be substitutable in PLLs and the related phosphotriesterase (PTE) family^1,2^. Because we used cobalt containing buffers during the enzyme purification, we investigated the chemical nature of the bound metals by X-ray anomalous scattering at a higher energy than the Co-Kedge (7.7089 keV). Bijvoet difference Fourier maps reveal one strong peak for each metal cation above the Co-K edge. This results suggests that the bi-metallic active site may contain cobalt, but not other common metal cations identified in similar enzymes such as zinc (Zn-K edge is 9.6586 KeV) or nickel (Ni-K edge is 8.3328)^1,2^. This result contrasts with known enzymes from the MLL family that were described to possess two zinc cations in their active site^3–5^.

This result also does not unambiguously determine the presence of cobalt in the active site, as peaks could also be explained by the presence of iron (Fe-K edge is 7.1120 KeV), or manganese (Mn-K edge is 6.5390 KeV). While the related phosphotriesterases (PTEs) enzymes and PLL lactonases were shown to be able to substitute their metals for Mn^6–8^, Fe was found to be present in PTEs’ and lactonases’ active sites without adding it to the buffers^1,9,10^. In the structures presented here, cobalt was modelled in the active site of AaL. The fact that these metal cations can often be substituted^1,7,9,11^ make the determination of the ‘real’ nature of the binuclear active site centers complicated, since it can be influence by the expression hosts, and downstream procedures including production, purification and crystallization conditions.


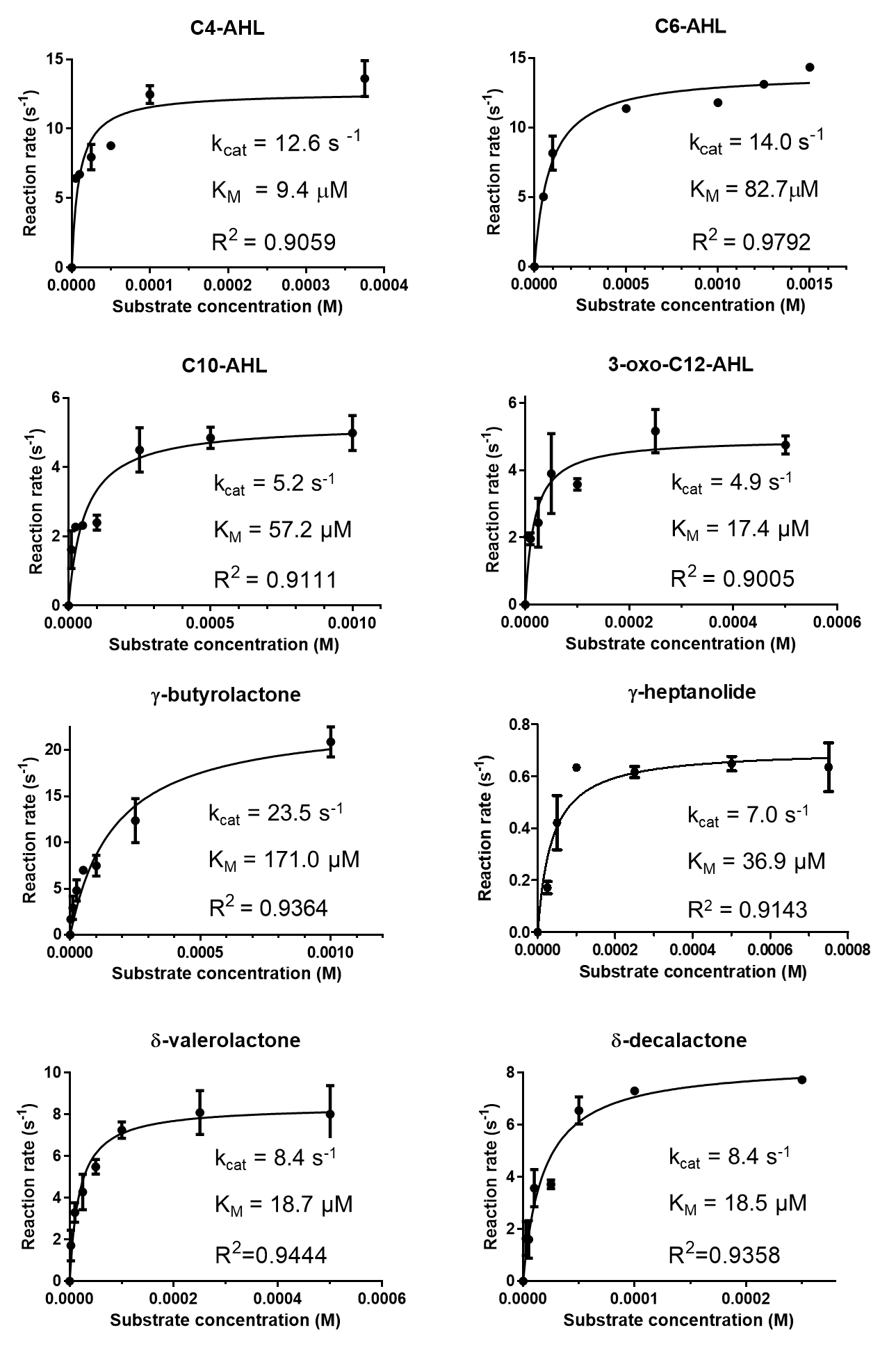


**Figure S3**: **Michaelis-Menten fitting of collected kinetic data for the lactonase AaL**. Kinetic data were fitted to the Michaelis-Menten equation for various substrates: AHLs (C4-, C6-, C10-, 3-oxo-C12-AHL) and oxonolactone (γ-butyrolactone, γ-heptanolide, δ-valerolactone and δ-decalactone). All measurements were performed in triplicate.


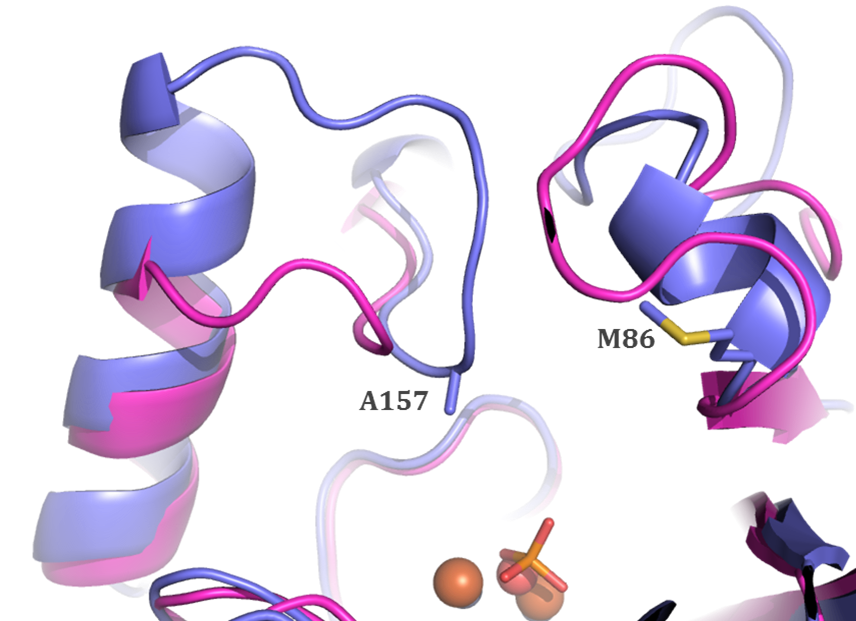


Figure S4: Difference in the active site accessibility of AaL and AiiA. The structure of AaL (in blue) shows a longer helix (left side) due to an insertion of seven amino acids (Y149 to E155) as compared to AiiA. Moreover, AaL exhibits a smaller M86-loop (right side) as compared to AiiA due to a deletion of one amino acid (T67). These differences result in a different binding cavity in the two enzymes.


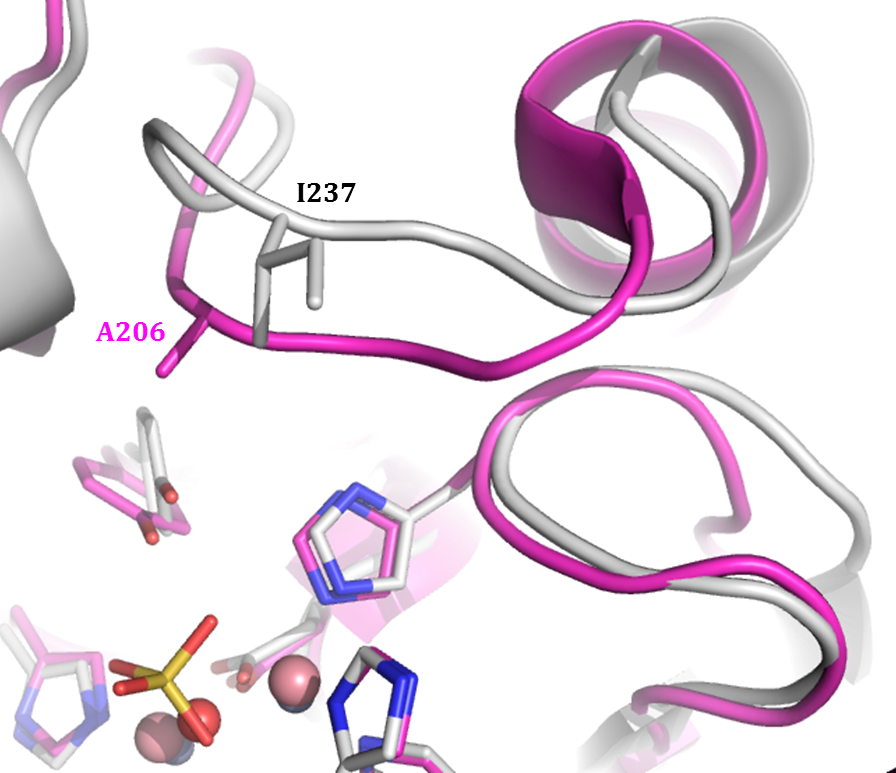


Figure S5: Overlay of the active sites of phosphate-bound structure of AaL (grey) and AiiA (pink) structure. Conformation of the 237-loop in AaL and the corresponding loop in AiiA (A206).


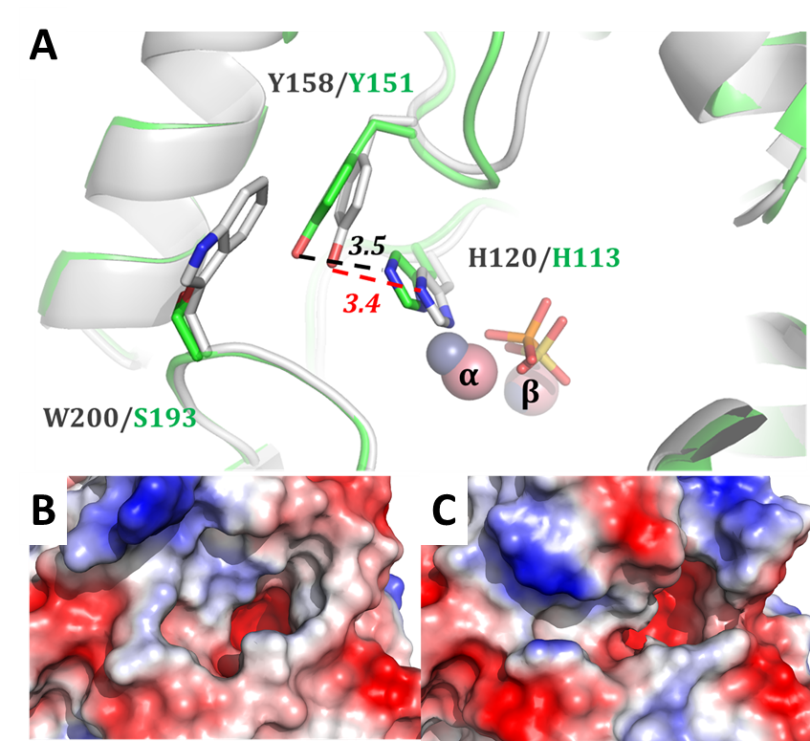


Figure S6: Differences between the active site of AaL and the active site of its closest relative AiiB (A) Active site residues and metal coordination in the phosphate-bound structures of AaL (grey sticks and pink spheres) and AiiB (green sticks and grey spheres). The distance between metals cations, not shown for figure clarity, are 3.5Å and 4.2Å, for AaL and AiiB, respectively. Vacuum electrostatic potential mapped on the surface of AaL (B) and AiiB (C) as calculated by PyMOL v1.8.0^12^.


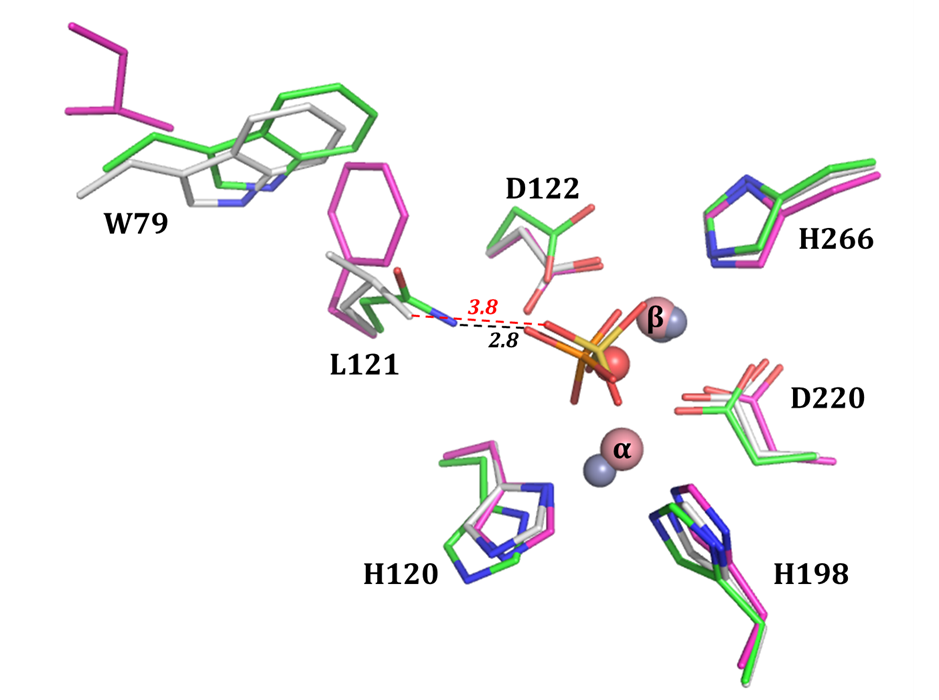


Figure S7: Overlay of the active sites of AaL (grey sticks), AiiA (pink sticks) and AiiB (green sticks). Residue L121 (in AaL) is located at 3.8 Ǻ distance from the closest oxygen atoms of the bound phosphate molecule, while the corresponding residue in AiiB (N114) forms a hydrogen bond with the bound anion. Metal cations are shown as pink spheres (AaL) and grey spheres (AiiB). Cations of AiiA are shown as light grey spheres.


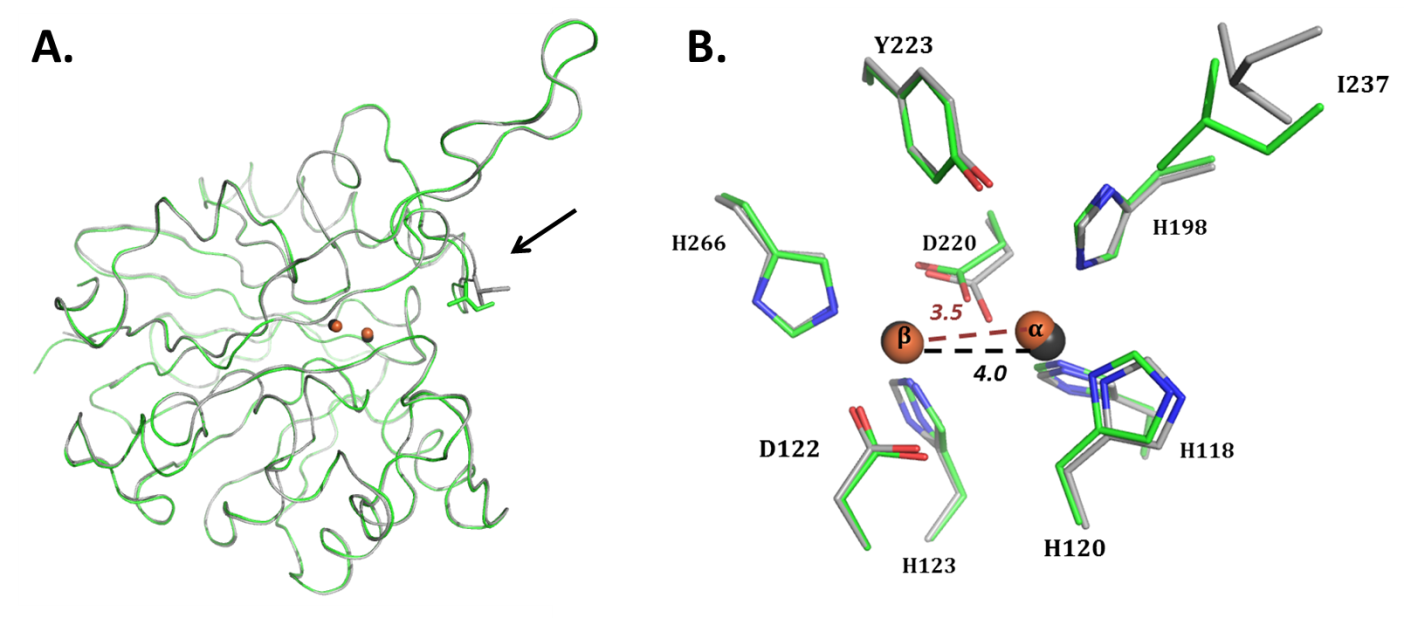


Figure S8: Comparison of the bound to phosphate (green) and bound to glycerol (grey) AaL structures. (A) Superposition of both structures. The residues I237 in green sticks for phosphate structure and grey sticks for glycerol structure shows different conformations. (B) The metal cations of the phosphate- bound structure (orange spheres) are separated by 3.5 Å whereas the distance between metal cations (black spheres) is 4.0 Å in the glycerol-bound structure. Rearrangement of metal coordination alters the positioning of other active site residues, including H120, H123, H266 and D220).


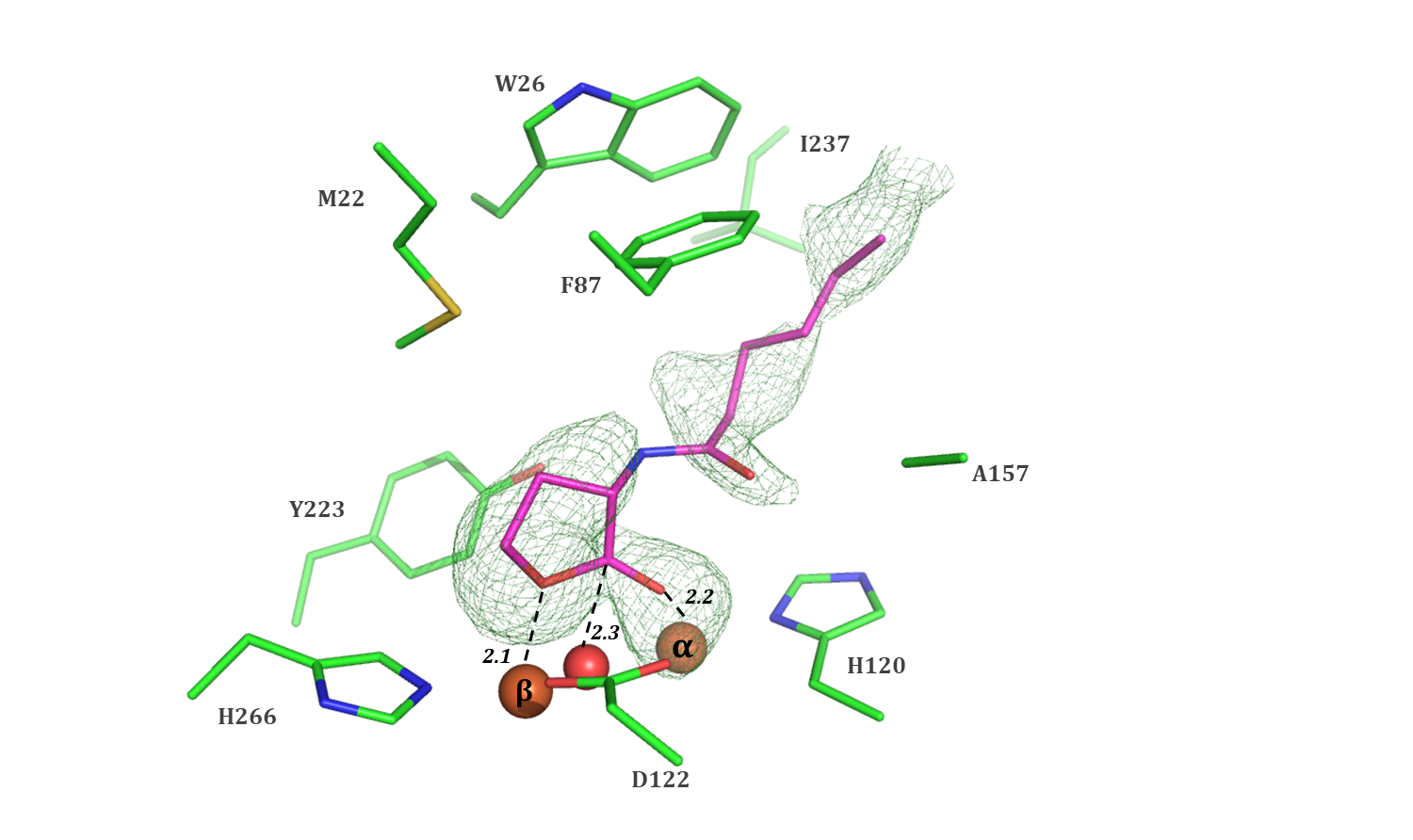


Figure S9: Electronic density for the C6-AHL complex structure (purple sticks). Fourier Difference map (Fo-Fc) (green mesh) calculated by omitting the ligant from the refinement calculations is contoured at 2.2 σ. Occupancy of the C6-AHL has been set to 0.7. Active site residues (green sticks) are shown and interactions between the bound C6-AHL, metal cations, and the bridging water molecule are highlighted as black dashes and distances are indicated in Ångstroms.


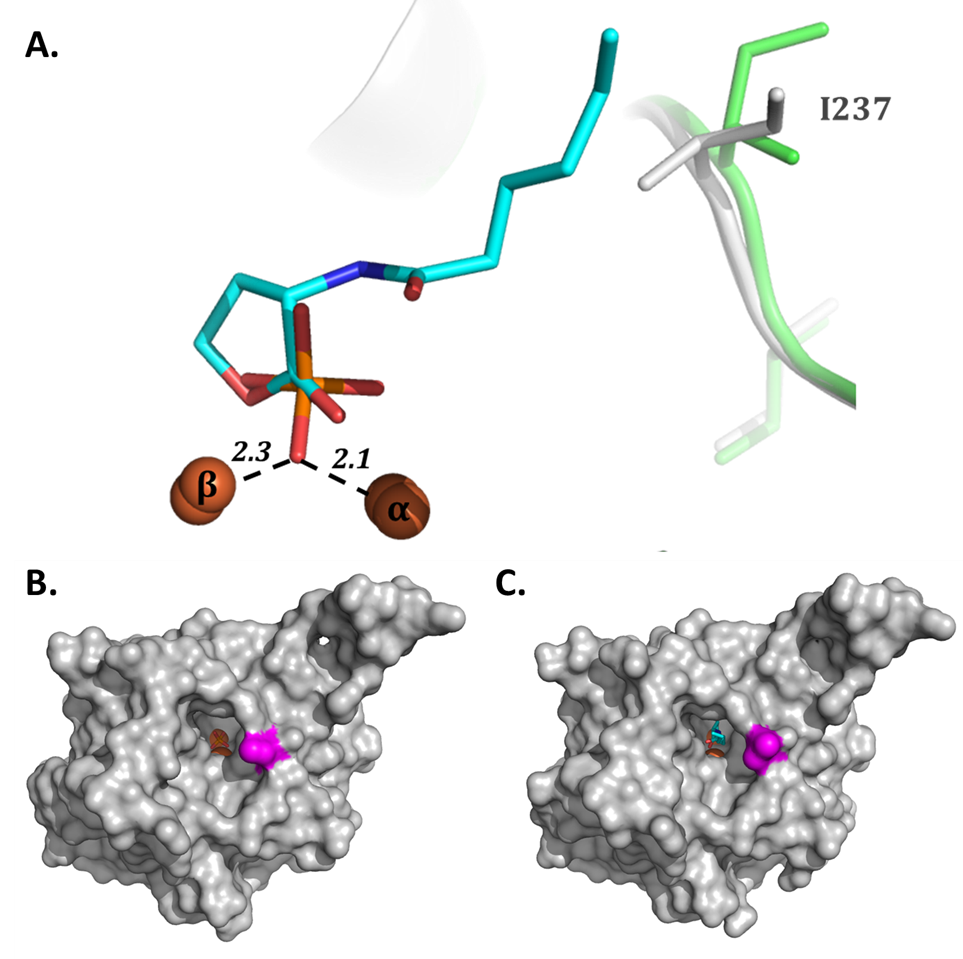


Figure S10: (A) Superposition of the phosphate-bound (grey sticks) and C6-AHL bound (green sticks) AaL structures. Phosphate molecule (orange sticks) and C6-AHL (cyan sticks) are shown. Residue I237 adopts slightly different conformation upon the binding of the AHL. (B) Accessibility to the active site in the phosphate-bound structure of AaL structure is slightly reduced by the conformation of I237 (purple), as compared to the C6-AHL (cyan) bound structure (C).


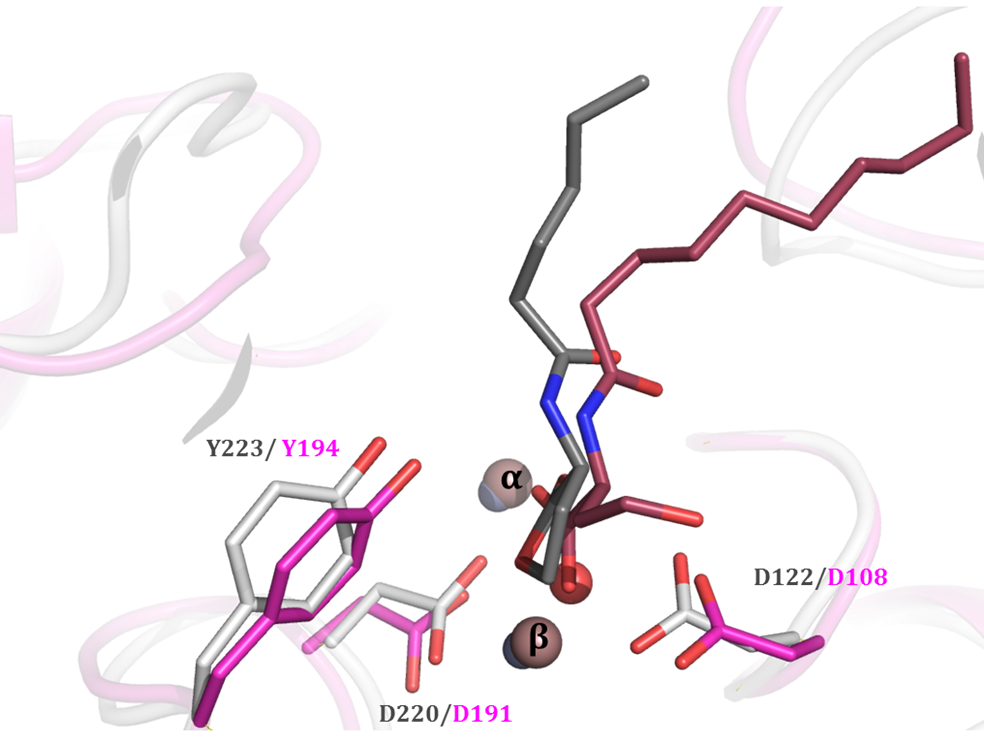


Figure S11: Overlay of the C6-AHL-bound AaL structure with the hydrolyzed C10-AHL-bound AiiA mutant F107W structure (PDB: 4J5H). Shown are AaL structure (light grey sticks) bound to a C6-AHL (dark grey sticks) and the AiiA F107W structure (magenta sticks) bound to a *N*-decanoyl-L-homoserine lactone hydrolytic product (dark pink sticks). The alcohol group of the *N*-decanoyl-L-homoserine product is oriented to residue D108. Metal cations are shown as spheres (pink and grey, for AaL and AiiA respectively). Putative catalytic water molecule is shown as red sphere.


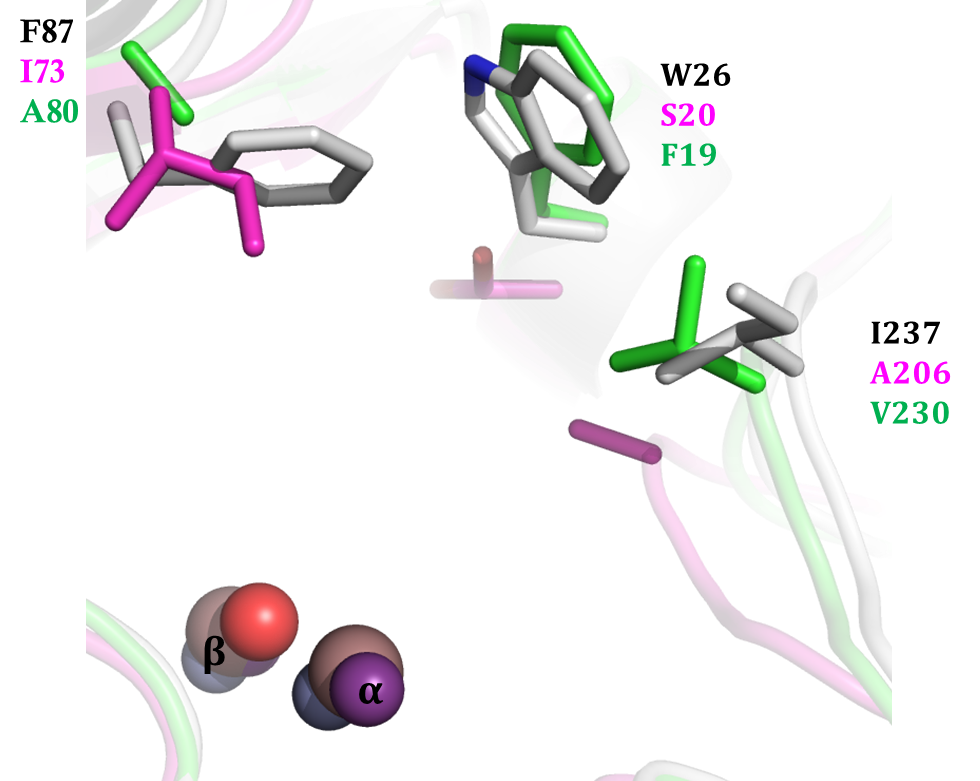


Figure S12: Overlay of the active sites of AaL (grey sticks), AiiA (pink sticks) and AiiB (green sticks). Residues involved in the hydrophobic patch of AaL are highlighted, as well as the corresponding residues in AiiA and AiiB.


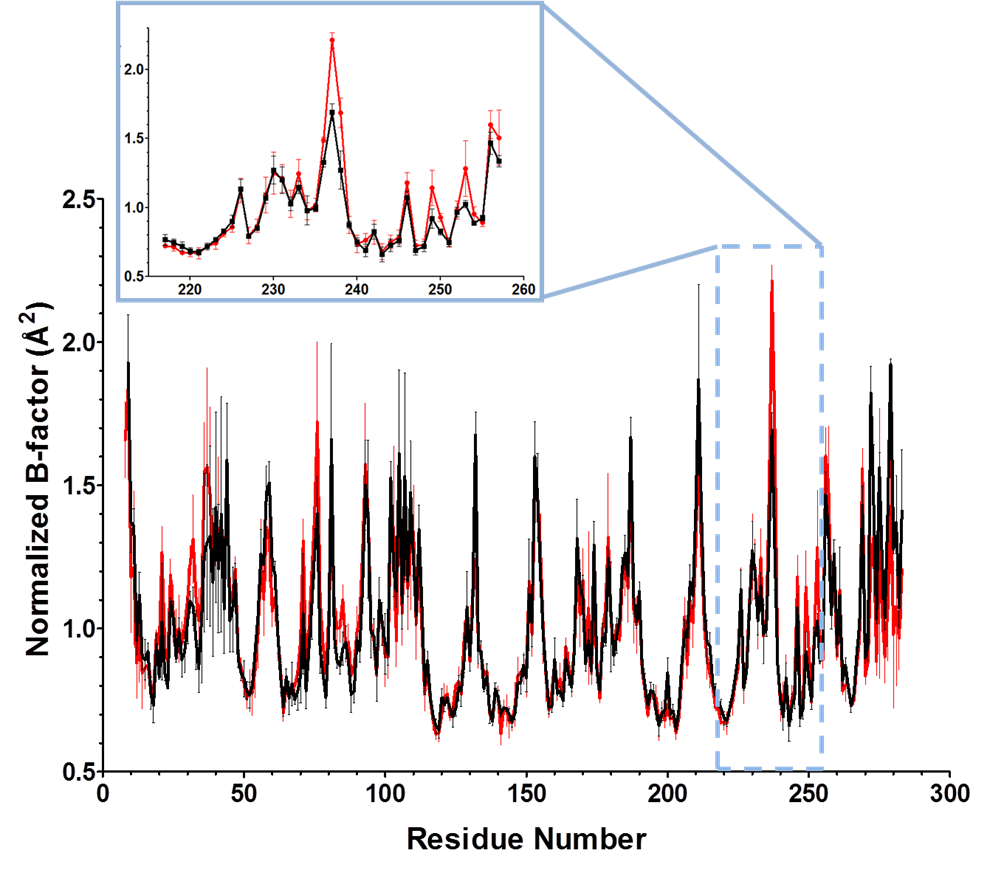


Figure S13: Comparison of normalized thermal B-factors of AaL structures bound to phosphate (red line) and C6-AHL (black line). The inset is highlighting the protein region around I237. The normalized B-factors shows that I237 shows the highest thermal agitation in the phosphate-bound structure. Nevertheless, I237 has a lower normalized B-factor in the C6-AHL bound structure, consistent with its interaction with the lactone molecule.

1. Elias, M. *et al.* Structural basis for natural lactonase and promiscuous phosphotriesterase activities. *J Mol Biol* **379,** 1017–28 (2008).

2. Omburo, G. A., Mullins, L. S. & Raushel, F. M. Structural characterization of the divalent cation sites of bacterial phosphotriesterase by 113Cd NMR spectroscopy. *Biochemistry (Mosc.)* **32,** 9148–55 (1993).

3. Kim, M. H. *et al.* The molecular structure and catalytic mechanism of a quorum-quenching N-acyl-L-homoserine lactone hydrolase. *Proc. Natl. Acad. Sci. U. S. A.* **102,** 17606–17611 (2005).

4. Liu, D. *et al.* Structure and specificity of a quorum-quenching lactonase (AiiB) from Agrobacterium tumefaciens. *Biochemistry (Mosc.)* **46,** 11789–11799 (2007).

5. Wang, W.-Z., Morohoshi, T., Someya, N. & Ikeda, T. AidC, a Novel N-Acylhomoserine Lactonase from the Potato Root-Associated Cytophaga-Flavobacteria-Bacteroides (CFB) Group Bacterium Chryseobacterium sp. Strain StRB126. *Appl. Environ. Microbiol.* **78,** 7985–7992 (2012).

6. Samples, C. R., Howard, T., Raushel, F. M. & DeRose, V. J. Protonation of the binuclear metal center within the active site of phosphotriesterase. *Biochemistry (Mosc.)* **44,** 11005–13 (2005).

7. Porzio, E., Di Gennaro, S., Palma, A. & Manco, G. Mn2+ modulates the kinetic properties of an archaeal member of the PLL family. *11th Int. Meet. Cholinesterases* **203,** 251–256 (2013).

8. Xue, B. *et al.* Structural evidence of a productive active site architecture for an evolved quorum-quenching GKL lactonase. *Biochemistry (Mosc.)* **52,** 2359–2370 (2013).

9. Jackson, C. J. *et al.* Anomalous scattering analysis of Agrobacterium radiobacter phosphotriesterase: the prominent role of iron in the heterobinuclear active site. *Biochem J* **397,** 501–8 (2006).

10. Chow, J. Y. *et al.* Directed evolution of a thermostable quorum-quenching lactonase from the amidohydrolase superfamily. *J Biol Chem* **285,** 40911–20 (2010).

11. Zhang, Y. *et al.* Enhancing the promiscuous phosphotriesterase activity of a thermostable lactonase (GkaP) for the efficient degradation of organophosphate pesticides. *Appl. Environ. Microbiol.* **78,** 6647–6655 (2012).

12. DeLano, W. L. The PyMOL Molecular Graphics System. *DeLano Sci. San Carlos CA USA* (2002).
